# Computer- and NMR-Aided Design of Small-Molecule Inhibitors of the Hub1 Protein

**DOI:** 10.3390/molecules27238282

**Published:** 2022-11-28

**Authors:** Atilio Reyes Romero, Katarzyna Kubica, Radoslaw Kitel, Ismael Rodríguez, Katarzyna Magiera-Mularz, Alexander Dömling, Tad A. Holak, Ewa Surmiak

**Affiliations:** 1 Department of Organic Chemistry, Faculty of Chemistry, Jagiellonian University, Gronostajowa 2, 30-387 Krakow, Poland; 2 Department of Drug Design, University of Groningen, A. Deusinglaan 1, 9713 AV Groningen, The Netherlands

**Keywords:** protein-protein interactions, anti-cancer therapy, nuclear magnetic resonance, protein-peptide docking, small-molecule inhibitors

## Abstract

By binding to the spliceosomal protein Snu66, the human ubiquitin-like protein Hub1 is a modulator of the spliceosome performance and facilitates alternative splicing. Small molecules that bind to Hub1 would be of interest to study the protein-protein interaction of Hub1/Snu66, which is linked to several human pathologies, such as hypercholesterolemia, premature aging, neurodegenerative diseases, and cancer. To identify small molecule ligands for Hub1, we used the interface analysis, peptide modeling of the Hub1/Snu66 interaction and the fragment-based NMR screening. Fragment-based NMR screening has not proven sufficient to unambiguously search for fragments that bind to the Hub1 protein. This was because the Snu66 binding pocket of Hub1 is occupied by pH-sensitive residues, making it difficult to distinguish between pH-induced NMR shifts and actual binding events. The NMR analyses were therefore verified experimentally by microscale thermophoresis and by NMR pH titration experiments. Our study found two small peptides that showed binding to Hub1. These peptides are the first small-molecule ligands reported to interact with the Hub1 protein.

## 1. Introduction

Human Hub1 (known also as UBL5; ubiquitin-like protein 5) is a small protein consisting of 73 amino acids that belongs to a large group of ubiquitin-like proteins. The yeast orthologue is called Hub1 and in mammals it is called beacon. Hub1s are widely expressed and are extremely conserved across phylogeny, and, despite a low residue similarity, they are homologues to ubiquitin [1,2,3]. Hub1, as other ubiquitin-like modifiers, was reported to interact with several target proteins. The mechanism of these interactions is unique and remains largely unexplored. There are two classes of the ubiquitin-like proteins [2]: one called ubiquitin-domain proteins (UDPs), consisting of proteins which harbor a ubiquitin fold within their polypeptide chain but do not form covalent conjugates, while the other group is ubiquitin-like modifiers (UBLs), which conjugate their target protein by covalent binding with an iso-peptide formation between their carboxyl at the C-terminus and the ε-amino groups of lysine residues of acceptor proteins [3]. Hub1, unlike other UBLs, does not have a characteristic di-glycine motif (GG motif) at its C-terminus. Instead, it has a di-tyrosine motif (YY motif), which seems to be crucial for the Hub1 function and its ability to conjugate with other proteins [4]. Furthermore, the Hub1 polypeptide chain lacks the exposed unstructured C-terminal tail, which is critical for other ubiquitin-like modifiers for their attachment. Since Hub1 appears to possess the features of both classes of ubiquitin-like proteins (UBLs and UDPs), it has been classified as a novel protein modulator that works by creating tight, non-covalent interactions with target proteins. Research by Mishra et al. [5] and Ammon et al. [6] shows that both yeast Hub1 and human UBL5 are involved in alternative splicing by non-covalent binding to HIND (Hub1-interacting domain) of the N-terminal domain of the Snu66 spliceosomal protein (small nuclear ribonucleoprotein). The alternative splicing of pre-messenger RNA diversifies gene products in eukaryotes and is guided by factors that enable spliceosomes to recognize a particular splice site. It is assumed that Hub1/UBL5 modifies the spliceosomes so that they can use certain noncanonical 5′ splicing sites [7]. Because Hub1 interacts with Snu66 on the opposite side to other ubiquitin-binding domains (UBDs), it makes this interaction unique [5]. Since malfunctions in alternative splicing can cause many diseases, such as hypercholesterolemia, premature aging, neurodegenerative diseases, and cancer, finding small-molecule antagonists that can inhibit Hub1/Snu66 interactions could not only allow the understanding of the mode of action of Hub1 in living organisms, but it may also start new approaches to anticancer therapy in the future [2,8].

Till now, there are no reports in the literature about small peptidomimetics or low molecular weight compounds that bind to Hub1 and inhibit the Hub1/Snu66 protein-protein interaction (PPI). Finding such agents for PPIs can be challenging [9,10]. This is because, compared with classical drug discovery against targets with well-defined binding sites, the PPI contact surfaces are frequently more extensive and comparatively flat, with most of the binding energy localized in “hot spots”. The Hub1/Snu66 interface displays such a fingerprint, embodied by weak scattered interactions around featureless surfaces [11]. 

In this study, we designed 14 peptides from the biologically active primary sequence of Snu66. We then carried out global docking with contact constrains and all atom reconstructions of the most accurate binding modes from the C-alpha representation. Analysis of the conserved molecular interactions on Hub1 allowed the delineation of the Snu66 side-chains to obtain a pharmacophoric model, which was validated by the microscale thermophoresis (MST) analysis. In parallel, we carried out a fragment-based NMR screening to find small-molecule compounds that can interact with the Hub1 binding site. The fragment-based drug discovery has become a mainstream approach to identify new chemical compounds that bind to medically important target proteins [12,13,14,15]. NMR is uniquely suited to screening compounds of low molecular weight and complexity (i.e., “fragments) [12,14,15]. Using rational approaches, such as structure-based drug design, the hit fragments are efficiently grown, linked, or fused into larger lead compounds that can be advanced into preclinical and clinical research [13,14].

Analysis of the activity of nearly 500 such fragments against Hub1 revealed the caveats to using NMR for protein-peptide interactions in proteins with pH sensitive amino acids located within a protein’s binding pocket. Our results demonstrate that basic and acidic amino acids create a local microenvironment that helps define a pK_a_ variable that drastically limits the hit identification by NMR. 

## 2. Results

### 2.1. Exploration of the Interface of Hub1/Snu66

The interface analysis was carried out with PDBePISA (Proteins, Interfaces, Structures, and Assemblies) server [16]. Briefly, we submitted the crystallographic coordinates of the PDB code of the Hub1 protein in complex with the Snu66 peptide (PDB ID: 3PLU). The server allows to retrieve information from the protein-protein interface, such as the solvation free energy gain (Δ_i_G) with its related P-value, the interface area, the surface, the symmetry operations to be applied to achieve in the formation of the interface, and the number of residues/atoms which form the interface itself. Furthermore, it allows to discriminate the residues involved in the interface from those that are exposed to the solvent, based on other parameters, such as the buried surface area (BSA), and type of interaction (hydrogen/disulphide/covalent bond or salt bridge). Negative values of Δ_i_G indicate that the protein-protein interface is mainly hydrophobic (entropic). 

As presented in Table 1, 20.3% (12) and 66.7% (15) of the Snu66 and Hub1 amino acids form an interfacing area of 509.1 Å^2^ and 483.1 Å^2^, respectively. An in-depth Δ_i_G analysis (Table S1), reveals the presence of a first group of ten residues with a positive energy contribution and a second one of two amino acids with a negative energy contribution: the former includes hydrophobic (Leu1, Ile3, Ile9, Leu13, Met15, Ile18 of Snu66), polar (Ser2, Thr6), and positively charged (Lys12, Lys16) side-chain residues while the latter, a positively charged chain residue (Arg10) and Pro17. We went further in our investigation by exploring the molecular interactions at the interface, as shown in Figure S1. As estimated by the Scorpion software, the interface is characterized by several hydrophobic interactions clustered primarily around Leu33, Val30, Lys29, and Asp26 of Hub1. This cluster includes eight interactions with Ile15, Leu19, and Met21 hydrophobic side-chains and a cooperativity binding score of 4.3 (total measured: 8.2). These results, along with the corresponding ΔiG values (1.48 kcal/mol, 1.01 kcal/mol and 0.95 kcal/mol) imply that this triad plays a strategic role in the Snu66 peptide binding to Hub1. Other van der Waals interactions are scattered downstream and involve Met1 and Lys17 side-chains of Hub1 which both interact with Ile9 and Ile24 Snu66. We also counted three hydrogen bonds, one between Lys17 of Hub1 and the amide CO of Snu66 Leu7 plus two between Asp22 (Hub1) and Arg16 (Snu66), and finally one ionic bond between Asp22 of Hub1and Arg16 of Snu66, whose cooperativity binding score (1.7) contributes only 21% to the total. With this information, we proceeded to design the peptide sequences and define the constraint of the molecular modeling.

### 2.2. Peptide Designe

For the peptide design, we removed one amino acid at a time until we obtained the smallest peptide with at least two of the three hydrophobic amino acids (Ile15 and Leu19) and Arg22 (Table 2). The importance of the Arg22 residue has been previously demonstrated by SK Mishra and co-workers [5], who observed the abolition of the Snu66 peptide binding to Hub1 upon Arg22 to the Ala 22 single point mutation. Shorter peptides were generated by removing one residue at a time starting at the C-terminal end (peptides 9–14). Finally, peptide 14 was designed by removing five and three amino acids from both ends with the aim of maintaining intact the alpha helix secondary structure.

### 2.3. Cooperativity Binding Network

The protein-protein molecular interactions analysis has been carried with Scorpion (DesertSci Ltd., Sydney, Australia). This software captures changes in the binding affinity of a small molecule or peptide because of a minor modification of the ligand structure. In addition, it extends the typology of non-covalent bonds and introduces a network approach to capture local cooperativity effects, thus allowing the intuitive identification of both key functional groups and hot spots on the protein surface. The quantification of the atomic contribution in the empirical scoring function (ScorpionScore) reflects how tight is the protein-protein (peptide) complex. This information can be found by submitting the crystal structure to the web server, free of charge at (https://desertsci.com accessed on 17 March 2022) and the results can be downloaded in pml format. Finally, from these files, we counted the peptide interactions on the Hub1 surface where the hot spots are clustered.

### 2.4. Peptide Docking

Upon completion of the definition of the amino acid sequences of the peptides, we pursued peptide docking with Hub1 and we evaluated the accuracy of our models, according to the CAPRI criteria (Table 3). Initially, the CABS-dock simulations generated 10,000 initial models in the CA representation which represented the initial large pool of conformations (Figure S2). A detailed overview of the accuracies and CABS-dock score of all peptides along with the model identifier, is presented in Table S3 while the final CAPRI ranking is given in Table S4.

During the first stage in the CA representation, none of the peptides meet the required parameters to be classified as at least “acceptable”, since they miss the atomic coordinate of the side-chains and beta carbons. This is reflected in the fact that all calculated values of Fnat are equal to 0 (Table S5). Nevertheless, a gradual rise of the accuracies of the models was observed. The data presented in Table 4 show a gradual increase in IRMS with 13%, 33% to 67% of the peptides of medoid, filtered and lowest groups below 1.0 Å. Furthermore, as can be seen in Table S6, that a similar trend was noticed in terms of LRMS: 73% of the “filtered” peptides were below the threshold of 2.0 Å. However, this fraction increased to 87% in the “lowest” peptides group. These results indicate that during the CABS-dock modeling, the method can produce models meeting two out of the three requirements to rank the models as “medium”. In addition, the top 10 medoid models yielded slightly worse accurate models (Table S7).

As for the second stage in the AA representation, we used ca2all.py, a Python script to reconstruct the CABS-dock models with side-chains and beta carbons. All peptides in the medoid group meet the parameters to be considered “acceptable” while those of “filtered” group were ranked as “medium”. In addition, ten peptides of the “lowest” group achieved the same evaluation, whereas the remaining five (i.e., 3, 5, 8, 11, 14) were judged “acceptable”. Note that 93% and 67% of the “lowest” group of peptides display indeed better accuracies than the “filtered” group, but only 67% exceeds the desirable threshold for the model to be classified as “medium”. These results mean that the “filtered” group possesses the necessary characteristics of accuracy and preservation of the native counts of the subsequent Hub1 hotspot analysis.

### 2.5. Binding and Hot Spot Analysis

**Figure 1:**
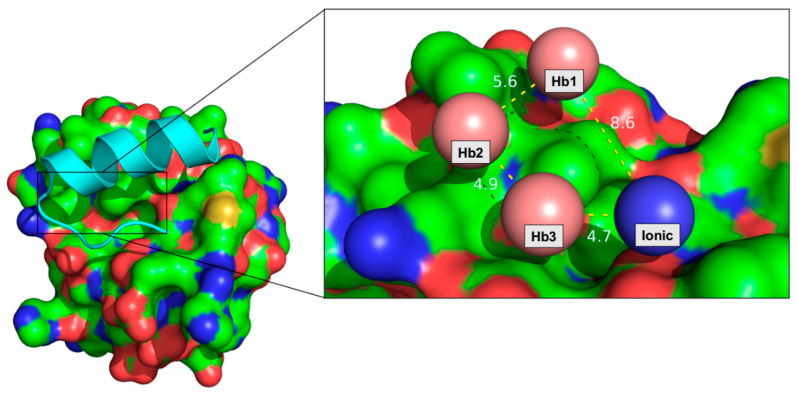
Crystal structure of Snu66-Hub1 (PDB ID: 3PLU). Snu66 is displayed as cyan cartoons while Hub1 as a colored surface (carbon: green, sulphur: yellow, oxygen: red, nitrogen: blue). The enlargement view shows the pharmacophoric model consisting of three hydrophobic (salmon spheres) and one ionic features (blue sphere). Distances between the centroids are illustrated as yellow dotted lines.

To select the pharmacophoric features of small molecules, we rationally classified the typologies of interactions at the protein peptide interface. We also counted the number of occurrences on a well-defined residue of Hub1 for each peptide. Next, we traced back the closest side-chain responsible for that specific cluster of interactions. A summary of both the cooperativity binding scores and hot spot residues are presented in Figure 1 and Table 5. 

On the one hand, the cooperativity binding score ranged from an estimated maximum of 9.35 for peptide-8 (IREKLGMKPI) to a minimum of 0.68 for peptide-14 (TNEIREKLGM) with no apparent linear correlation with the primary sequence length. In a few cases, we observed a decrease following the removal of a specific side-chain, as in the case of peptide-1 (SIEETNEIREKLGMKPI). Additionally, as exemplified by peptide-2 (IEETNEIREKLGMKPI) and peptide-7 (EIREKLGMKPI), the simultaneous removal of two or more amino acids from the N-terminal end resulted in either no or a major change in the cooperativity binding network score. When we went to remove the amino acids from the C-terminus end, the peptides with between 5 and 10 residues in length tend to have an average of 4.93 ± 1.39 with similar scores between the longest (peptide-8, IREKLGMKPI) and the shortest (peptide-13, IREKL) peptides. Interestingly, a drastic drop was noticed in peptide-14 when the first five amino acids upstream and the last three downstream of the Snu66 full-length sequence are removed, at the same time. Similarly, peptide-11 exhibits a reduced cooperative binding network, compared to the Snu66 peptide. A possible explanation for these results might be the loss of residues crucial for anchoring the peptide on the surface, e.g., Ile-24 and/or Ile-9. On the other hand, the top five hot spots of Hub1, accounting for the 68% of the interactions estimated by the Scorpion software, consist of Val-30, Asp-26, Asp-22, Leu-33, and Lys-29. The corresponding Snu66 residues converging on these hot spots are Ile-15, Arg-16, Leu-19, and Met-21. Accordingly, our final pharmacophoric model consists of three hydrophobic features and one positively charged feature at the distances of 5.6 Å, 4.9 Å, 4.7 Å, and 8.7 Å each from the respective centroids. The resulting model thus rationalized was then subsequentially assessed via NMR and MST experiments.

### 2.6. Peptide NMR Analysis

**Figure 2:**
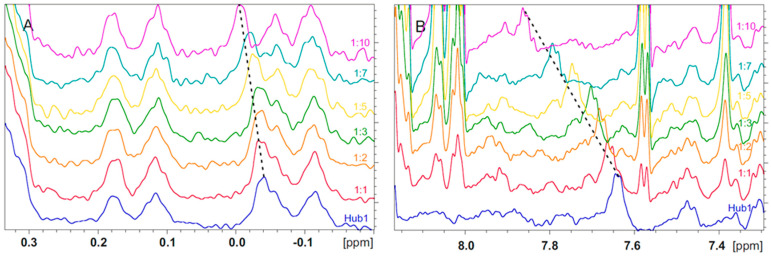
^1^H NMR titration experiment of Hub1 with peptide-14. (**A**) Aromatic region of the spectra (**B**) Aliphatic region of the spectra. Color coding: reference Hub1—blue, 1:1 ratio of Hub1: peptide-14—red, 1:2 ratio of Hub1: peptide-14—yellow, 1:3 ratio Hub1: peptide-14—green, 1:5 ratio Hub1: peptide-14—orange, 1:7 ratio Hub1: peptide-14—light blue, 1:10 ratio Hub1: peptide-14—purple.

We next assessed the binding affinity of peptides-11 and 14 using NMR. For that purpose, we titrated the Hub1 protein with peptides-11 and 14 using a ratio from 1:1 to 1:10 and measured the ^1^H NMR spectrum of each solution (Figure 2). The analysis of the superimposed spectra (Figure S3) showed in both cases that there are changes in chemical shifts in the aliphatic (δ = 0.04 ppm) and aromatic (δ = 6.5 ppm) regions, though larger for peptide-14. Changes in the buffer pH after titration were relatively small, being 6.7 and 6.5 for peptide-11 and 14, respectively, which seems to be not relevant and suggests a weak interaction between the peptides and Hub1 instead.

### 2.7. Microscale Thermophoresis Analysis

**Figure 3:**
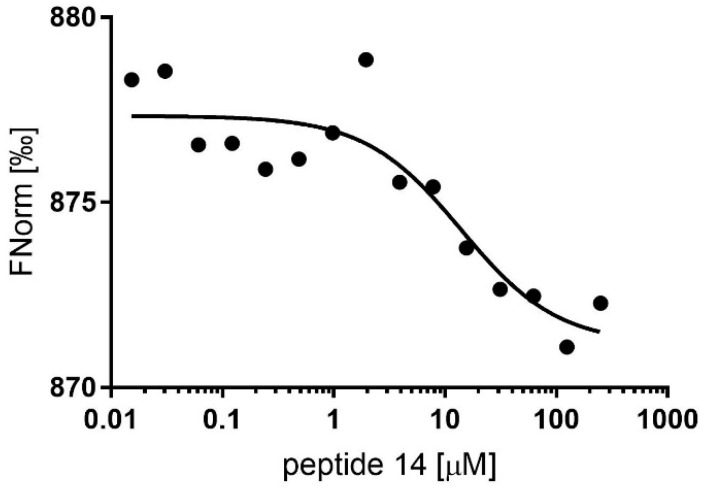
MST analysis of the binding of peptide-14 to Hub1.

To confirm our findings, we decided to perform microscale thermophoresis (MST)-based experiments for peptides-11 and 14. For peptide-14, we obtained a typical sigmoid curve, and the dissociation constant (K_D_) was 14.04 ± 8.3 μM (Figure 3). In contrast, we detected no significant binding events for peptide-11 (Figure S4). Overall, these data show the binding of peptide-14 with Hub1 in the μM range. 

### 2.8. NMR Screening of the Small Molecules

**Figure 4:**
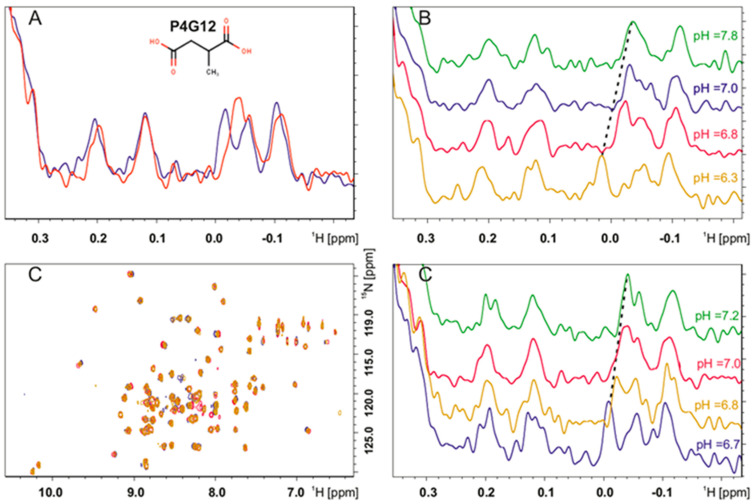
The NMR experiment of the Hub1 protein titrated with small molecular fragments. (**A**) ^1^H NMR aliphatic spectrum of reference Hub1 (red) superimposed with the spectrum of Hub1 titrated with 10−fold excess of P4G12 (blue) indicating a possible interaction of compound P4G12 with the Hub1 protein; (**B**) pH dependence of the aliphatic range of ^1^H NMR of the Hub1 protein indicating the sensitive nature of Hub1 to even small pH changes: blue—initial pH of the protein in PBS, red—pH = 6.8; yellow = 6.3; green = 7.8; (**C**) pH correction of Hub1 with the P4G12 small fragment. **Left corner**—^1^H-^15^N HMQC spectra; **right corner**—aliphatic ^1^H spectrum, red-reference Hub1, blue—after 1:10 addition of P4G12, yellow—pH corrected to 6.8, green—pH corrected to 7.2. Recorded spectra have shown that all changes visible during the titration of Hub1 by P4G12 can be reversed by even small pH changes.

Following the peptide docking analysis, we performed an experimental fragment-based screening of a library of small molecules using NMR techniques (Tables S8–S12). This library consists of the typical building blocks used in the multi-component Ugi and Passerini reactions, allowing for the rapid discovery of readily expandable hit fragments with a wide chemical diversity. To increase the throughput faster, in the first step, the ^1^H NMR spectra of the Hub1 protein treated with a 10-fold molar excess of the five fragments cocktail were recorded. When the perturbations in the chemical shifts of the aliphatic region were observed, we examined the possible binding with single compounds using the ^1^H-^15^N SOFAST-HMQC (band-selective optimized flip-angle short-transient heteronuclear multiple quantum correlation). To achieve this, the protein was titrated with a stock solution of small fragments in DMSO (dimethyl¬sulfoxide) with a 10-fold molar excess of the compound (Figure 4, Table S13). On this basis, 31 fragments were identified as potential hits. From this number of initial fragments, 84% are composed of carboxylic acids, 20% of which are linked to an equal functional group. Regardless of the number of spacer atoms, 11 of these are linked to the aromatic ring (e.g., P4H4, P4G9 and P1E9) and only eight to the hydrophobic alkyl chain (e.g., P4H12, P4H9 and P4H8). When comparing these results with our proposed pharmacophoric model, some of the fragments from the same plate, for example P4H4, P4G9, and P4H9, meet three out of the four requirements that we rationalized. However, it would be desirable to replace the negatively charged carboxylic group at pH 7.4 with a positive group, such as a primary amine, hydrazine, or amidine. The similarity of the selected structures, especially their acidity, prompted us to analyze the possibility of the influence of pH on the Hub1 protein during titration. The changes in pH during titration were relatively small, such as those of the tested peptides, that were in the range of approx. 0.3–0.4 lower than the initial pH. Such differences typically should not affect the chemical shifts in the aliphatic region of the NMR spectrum and all perturbations should result from the binding interactions. To confirm this thesis, we adjusted the pH of the Hub1 solution to the experimental conditions without the added compounds and it turned out that the protein is highly sensitive to pH changes (Figure 4B). This sensitivity does not indicate that the results obtained are solely due to changes in pH, as the binding may be independent of it. To investigate what causes the perturbations in the chemical shifts, we adjusted the pH of the protein titrated with small molecular fragments to the initial one (Figure 4C—P4G9—2-hydroxy-2-phenylacetic acid; pH of 6.8 after 1:10 ligand addition, corrected to pH of 7.2; Figure S5—P4G12—2-methylsuccinic acid; pH of 6.7 after 1:10 ligand addition, corrected to pH of 7.2). Unfortunately, we observed the reversing of changes in the spectra during the pH correction, which leads to the conclusion that the perturbations of the chemical shifts may be related to the acidic nature of the compounds rather than to their interaction with the protein.

## 3. Discussion and Conclusions

When the protein interface is large and flat, the general strategy for translating peptides into small molecules consists of deleting, shortening, substituting, and alanine scans of amino acids one at a time [11]. This step aims to identify the smallest peptide fragment essential for preserving the biological activity. The side-chains can then be replaced with non-peptide scaffolds to mimic the interaction with the protein. The scheme described herein may encounter barriers, such as in case of flat, hydrophilic protein interfaces without a deep cavity into which amino acid anchors can fit, thus making the translation process difficult and with no guarantee of success. The Hub1/Snu66 interface displays such features, embodied by weak, scattered interactions around featureless surfaces [9]. Thus, in view of the advantages and drawbacks of this strategy, the docking methodologies can be useful for predicting the binding of an engineered peptide. These methodologies are classified in local (e.g., DynaDock [17], HADDOCK [18], global (e.g., PIPER-FlexPepDock [19], CABS-dock [20], ClusPro PeptiDock [21]) and template-based (e.g., GalaxyPepDock [22], PepComposer [23]), and may be assisted by other tools for the coarse refinement of the peptide-protein models [24] or the prediction of the peptide/binding site [25]. 

Complementary to the docking analysis, an emerging method in drug discovery is the fragment-based screening [26,27]. This approach allowed access to a completely new area of the chemical space, which is the greatest advantage of fragment screening over the high-throughput screening (HTS) that has been widely used over decades and is based on the preexisting compound sets. Furthermore, the fragment-based screening requires smaller libraries, compared to HTS, and higher hit rates are observed. It relies on identifying the chemical fragments defined by the ‘rule of three’ consisting of molecular weight < 300, cLogP ≤ 3, number of hydrogen bond donors and acceptors ≤ 3. These fragments generally display weak affinities towards the biological target, implying that they must be screened at high concentrations for a proper identification.

Nuclear magnetic resonance (NMR) techniques are often used for the fragment-based screening [28,29,30]. In comparison with other screening methodologies, NMR can detect the binding of small-molecule compounds to macromolecular targets over an extraordinary broad affinity range from covalent to millimolar. A unique feature of NMR is its robust capability to detect weak intermolecular interactions, which are extremely important in fragment-based screening [31,32,33,34,35]. With the application of this method, it is also possible to have structural information that can identify the binding site or binding modes of the fragments [26,34,36]. 

To identify small molecule ligands for Hub1, we have used interface analysis, peptide modeling, MST analysis, and NMR screening. As a first step, we decided to perform an interface analysis of the interaction between Hub1 and the HIND domain of the Snu66 protein to better understand the desirable profile of ligands that would interact with the Snu66 binding site of Hub1. Two peptides were selected and their Hub1 affinities were tested by NMR and MST analysis. These methodologies both confirmed the binding between the selected peptide-14 to the Hub1 protein, in contrast peptide-11’s binding is weaker but clearly visible in the NMR analysis. The observed changes in the NMR spectrum are not globally abundant in the spectrum, which can indicate a weak ionic interaction between the peptide and aspartic acid in the protein binding pocket (Figure S6). Such small shifts can also be caused by changes in the pH that affect the charge of the amino acids that are crucial for binding, especially when the peptides themselves have charged amino acids. 

With this knowledge in hand, we extended our research to find small-molecule non-peptide fragments that bind to the Hub1 protein. We carried out the NMR ^1^H-^15^N SOFAST-HMQC titration of the fragment’s library. Our results showed that all experimentally selected “hits” were acidic in nature and the perturbations in the chemical shifts were most likely caused by the change in pH. The Hub1 protein is sensitive to small changes in pH, and therefore perturbations in the NMR signals in the aliphatic region, which shows mostly the protein side-chain methyl groups, may be due to changes in pH, although the weak binding of the fragments to Hub1 cannot be excluded. The situation we have observed, although infrequent, probably results from the specific nature of the Snu66-binding pocket of Hub1. The peaks in the aliphatic region of the NMR spectra originate from the methyl side-chains of leucine, isoleucine, alanine, or other aliphatic residues, therefore, should be resistant to the pH changes of the solution. There may be times, however, that the proximity of the pH-sensitive amino acid, such as arginine, may influence changes even in the aliphatic region of the NMR spectrum. The Snu66 binding pocket of Hub1, is widely occupied by pH-sensitive residues (especially Lys29, Asp22, Asp26) which are close to the aliphatic amino acids (Leu33 or Val30). Given this possibility, the NMR methodology turned out to be insufficient to unambiguously search for the fragment binding to the Hub1 protein. Positive hits in the NMR experiments for this protein have to be verified by another (orthogonal) method. However, NMR can be used to exclude compounds that do not interact with the protein. If changes in the NMR spectrum of a protein are not observed during the ligand titration, the respective compounds will not interact with the protein. To sum up, we have shown that NMR may not be the only method used to indicate hits in this particular case, but may be useful for excluding fragments that do not bind to the Hub1 protein. Note also that the distance between the pharmacophoric features of our proposed model implies a molecule of at least more than three carbon-carbon bonds. This certainly violates one of the ideal rules of a fragment, or “rule-of-three”, and implies that future screening efforts must consider other alternatives to occupy at least three of the four pharmacophoric sites. One possibility might be macrocyclic peptides.

Our study found that two small peptides that showed a weak affinity for binding to Hub1. These peptides are the first small-molecule ligands reported to interact with the Hub1 protein. We recognize this as the starting molecule for further optimization and discovery of a new Hub1 ligand.

## 4. Materials and Methods

### 4.1. Peptide Docking Methodology

Molecular modeling was performed with CABS-dock (C-B and Side group). This methodology consists in the coarse-grained docking simulation searching for the peptide binding site while enabling for the full flexibility of the peptide itself and the fluctuations of the protein backbone. 

The conformational sampling employs the replica exchange Monte Carlo. Each ensemble is the result of 20 Monte Carlo temperature annealing cycles per each input peptide (see the supporting information for more detailed commands), each including 50 Monte Carlo cycles, thus producing a total of 1000 printed snapshots. CABS dock employs a knowledge-based force field including the potential parameters from the structural regularities observed in the protein crystal structures. More detail of the refined force field and its improved accurate modeling are extensively covered in its original publication [37]. We conducted for each of the 15 peptides ten independent simulations, thus producing 1000 models. The lowest energy models from each trajectory converged into a selection of 1000 top-scored models. 

Following the structural clustering, ten sets of similar models are generated from which ten cluster representatives (medoids) are retrieved for the final reconstruction in all atom representations. The clustering methods employs the k-medoid algorithm, a fast and convenient algorithm less sensitive to outliers, and RMSD was used as the structural similarity parameters to evaluate the docking poses adopting similar binding modes and conformation. the default parameters were used for clustering (e.g., 10 medoids in the k-medoids clustering algorithm “-k NUM, -clustering-medoids NUM”, 100 iterations in “-clustering-iterations NUM”).

The scoring module of CABS dock ranks the representative k-models, based on energetic and structural similarity criteria. By default, 1000 low-energy models from the trajectories are clustered. This procedure enables to retain the best poses for the subsequent evaluation during the initial filtering. Moreover, a different model selection, based on the cluster density values is adopted to rank the top ten medoids in all atom representations [38]. 

We grouped the possible candidates into three groups, i.e., lowest, filtered and medoid, corresponding to lowest RMSD models with CABS-dock energy from the ten trajectories and cluster representatives. A detailed summary of the input commands can be found in Table S2 [37,39,40,41]. For our work, we employed the Python standalone version [20] which incorporates additional modules, such as clustering, scoring, data plotting, and the re-construction of all-atom models from the CA representation with Modeller. The secondary structures were assigned with DSSP [42,43]. Moreover, we employed a licensed version of Modeller (v10.1) for the optimization of the receptor structure within the top 10 models [42] a 2all.py Python script was used to create the all-atom representation from the best and lowest filtered models using the command ca2all.py -i CA_model.pdb -o AA_model.pdb. For the calculation of the CAPRI criteria, we used the crystallographic coordinates of the Snu66 peptide in complex with Hub1 (3PLU) and its apo form (1M94) from the protein data bank (PDB) and we treated these structures as receptors and reference control, respectively. Finally, the molecular interactions between the refined pose and the receptor were generated with Scorpion. All figures were rendered with Pymol version 2.3.4 (Schrödinger, LLC, New York, NY, USA).

### 4.2. Protein Purification

Hub1 (residues 1-73) was expressed in *E. coli* BL21 (DE3) (New England Biolabs) using the pET28a vector. The protein was either grown in LB or M9 minimal medium at 37 °C and was subsequently induced with 1 M IPTG (isopropyl β-d-1-thiogalactopyranoside) at OD_600_. The bacterial culture was grown overnight at 25 °C. The cells were then harvested by centrifugation (10 min, 5000× *g*); the obtained pellet was then dissolved in the lysis buffer containing 50 mM NaH_2_PO_4_, 30 mM NaCl, 10 mM imidazole at pH 8.0 and was subsequently disrupted by sonication (five times for 2 min using a macrotip, output control 8, 80%). The disrupted material was then centrifuged (15 min, 15,000× *g*) and applied to Ni-NTA (nickel-nitrilotriacetic acid) resin previously equilibrated with a lysis buffer; the column was then washed with buffer containing 50 mM NaH_2_PO_4_, 300 mM NaCl, 20 mM imidazole at pH 8.0, and the protein were eluted with the buffer containing 50 mM NaH_2_PO_4_, 300 mM NaCl, 250 mM imidazole at pH 8.0. The last purification step consisted of the size exclusion chromatography (SEC) using a Superdex 75 column (Akta Pure, GE Healthcare, Chicago, IL, USA) using PBS buffer at pH 7.4.

### 4.3. Microscale Thermophoresis 

Hub1 buffered in 20 mM HEPES, pH 7.5, 100 mM NaCl, 0.05% Tween-20 was labeled using His-Tag Labelling Kit RED-tris-NTA 2nd generation (MO-L018, NanoTemper, Munich, Germany), according to the manufacturer’s protocol. The labeled Hub1 was titrated with serial dilutions of peptide-11 or peptide-14, ranging from 0.0015 µM to 250 µM. The final concentration of Hub1 was 50 nM. All measurements were calculated on a Monolith NT.115 instrument (NanoTemper, Munich, Germany) using the standard capillaries at the excitation power of 60% and the medium MST power in duplicates. The results were analyzed in GraphPad Prism software (GraphPad Prism version 8.0.0 for Windows, GraphPad Software, San Diego, CA, USA).

### 4.4. Nuclear Magnetic Resonance

The NMR measurements were carried out at 300 K on an ultrashielded 600 MHz Bruker AVANCE 3 (Bruker Corporation, Billerica, MA, USA) spectrometer equipped with a liquid nitrogen cryogenic system. Uniform ^15^N isotope labeling was achieved by the expression of the protein in the M9 minimal media containing ^15^NH_4_Cl as the sole nitrogen source. The NMR buffer for ^15^N-labeled protein was PBS pH 7.4 with 5 mM DTT (dithiothreitol). To provide a lock signal, 10% (*v*/*v*) of D_2_O was added to the samples. Water suppression was carried out using the WATERGATE sequence [44]. Stock solutions of the inhibitors used for titration were prepared in DMSO-*d*_6_. The binding of small molecule fragments towards Hub1 was measured with the 2D SOFAST-HMQC spectra [45] of the ^15^N-labeled Hub1 recombinant protein in 3-mm NMR capillaries. For the preliminary screening, the mixture of three compounds was added in a molar ratio compound to protein 1:10. Then the ^1^H and SOFAST-HMQC spectrum was recorded and compared with the reference spectrum of the protein Hub1 titrated with a suitable amount of DMSO. When any activity occurred, all compounds which were in the mixture were tested one by one. The individual spectra were recorded in 10-fold excess of the inhibitor either by ^1^H NMR or SOFAST-HMQC. The ^1^H spectra of Hub1 with peptides-11 and 14 (10 mM solution in water) were measured during a 9-step titration routine; SOFAST-HMQC were recorded for the 10-fold excess of the peptide. The spectra were processed with TopSpin 3.2 software (Bruker Corporation, Billerica, MA, USA).

## Figures and Tables

**Table 1:** Interface details estimated by PDBePISA.

	Snu66 Peptide		Hub1	
**Selection range**	C		A	
**Class**	Protein		Protein	
**Symmetry operation**	x, y, z		x, y, z	
**Symmetry ID**	1_555		1_555	
**Number of atoms**				
Interface	40	29.90%	55	9.40%
Surface	99	73.90%	351	60.00%
Total	134	100%	585	100%
**Number of residues**				
Interface	12	66.70%	15	20.30%
Surface	18	100.00%	69	93.20%
Total	18	100.00%	74	100.00%
**Solvent-accessible area, Å**				
Interface	509.1	31.10%	483.1	10.30%
Total	1636.5	100%	4700.6	100.00%
**Solvation energy, kcal/mol**				
isolated structure	−8.2	100%	−63.6	100%
gain on complex formation	−4.9	59.60%	−4.7	7.40%
average gain	−4	49.30%	−1.5	2.40%
***p*-value**	0.353		0.125	

**Table 2:** Overview table showing the peptides docked in the Hub1 protein including their primary sequence and the residue length.

ID	Sequence	Length
Snu66 peptide	LSIEETNEIREKLGMKPI	18
Peptide-1	SIEETNEIREKLGMKPI	17
Peptide-2	IEETNEIREKLGMKPI	16
Peptide-3	EETNEIREKLGMKPI	15
Peptide-4	ETNEIREKLGMKPI	14
Peptide-5	TNEIREKLGMKPI	13
Peptide-6	NEIREKLGMKPI	12
Peptide-7	EIREKLGMKPI	11
Peptide-8	IREKLGMKPI	10
Peptide-9	IREKLGMKP	9
Peptide-10	IREKLGMK	8
Peptide-11	IREKLGM	7
Peptide-12	IREKLG	6
Peptide-13	IREKL	5
Peptide-14	TNEIREKLGM	10

**Table 3:** CAPRI parameters to assess the peptide quality, IRMS (interface root mean square) LRMS (backbone root mean square) and Fnat (fraction native contacts).

Quality	LRMS (Å)	IRMS (Å)	Fnat
Acceptable	<5.0	<2.0	>0.2
Medium	<2.0	<1.0	>0.5
High	<1.0	<0.5	>0.8

**Table 4:** Summary table of IRMS values.

IRMS (Å)		CA Representation CABS—Dock Simulation			AA Representation ca2all.py		
ID	Length	Medoid	Filtered	Lowest	Medoid	Filtered	Lowest
Peptide-1	17	1.28	1.04	0.94	1.23	1.00	0.92
Peptide-2	16	1.04	0.90	0.94	0.95	0.75	0.78
Peptide-3	15	1.57	1.16	0.97	1.43	1.07	0.92
Peptide-4	14	1.29	1.23	1.03	1.16	1.13	0.88
Peptide-5	13	1.61	1.09	1.11	1.41	0.97	1.17
Peptide-6	12	1.53	1.17	1.03	1.46	1.27	0.90
Peptide-7	11	1.28	1.08	0.97	1.44	1.18	0.99
Peptide-8	10	1.50	1.16	1.03	1.34	1.20	0.92
Peptide-9	9	1.23	1.12	0.65	1.48	1.01	0.71
Peptide-10	8	2.07	1.00	0.84	2.08	0.89	0.84
Peptide-11	7	1.40	0.93	0.95	1.45	1.00	0.96
Peptide-12	6	0.92	0.57	0.86	0.94	0.88	0.76
Peptide-13	5	0.98	0.86	0.71	1.31	0.97	0.79
Peptide-14	10	1.57	0.84	0.90	1.50	0.89	0.88
Snu-66 peptide	18	1.95	1.24	1.09	1.70	1.09	1.00

**Table 5:** Summary of the binding cooperativity analysis including the molecular interaction count and total score for each individual peptide.

ID	Hot Spots Count										Cooperativity Score
	MET-1	ASP-26	LEU-19	VAL-30	LYS-29	ASP-22	LEU-33	LYS-17	CYS-18	GLU-21	
Snu66_peptide	2	6	1	6	2	4	1	0	1	1	8.35
Peptide-1	5	2	4	1	0	1	3	0	1	0	6.09
Peptide-2	2	2	4	2	1	2	1	1	3	0	6.79
Peptide-3	1	3	0	3	3	2	2	6	2	1	5.41
Peptide-4	0	6	4	4	3	2	6	0	0	1	8.15
Peptide-5	2	1	4	4	1	2	2	0	1	3	7.96
Peptide-6	5	5	1	2	2	3	1	2	3	1	9.35
Peptide-7	0	2	4	0	2	6	1	0	1	1	3.33
Peptide-8	2	5	2	4	4	2	2	1	1	2	5.85
Peptide-9	0	3	0	5	3	3	3	1	2	0	6.04
Peptide-10	0	0	0	5	2	2	1	0	1	0	4.42
Peptide-11	1	3	0	2	0	3	3	0	0	0	2.50
Peptide-12	0	0	2	2	3	2	3	0	0	0	5.18
Peptide-13	0	1	0	2	4	2	1	0	2	0	5.56
Peptide-14	0	3	0	2	0	3	2	2	1	0	0.68
**Total**	20	42	26	44	30	39	32	13	19	10	

## Data Availability

The datasets generated and analyzed during the current study are included in this published article (and its Supplementary Information files) or available from the corresponding author upon reasonable request.

## Supplementary Materials

The following supporting information can be downloaded at: https://www.mdpi.com/article/10.3390/molecules27238282/s1, Figure S1: Close up view of the molecular interactions between the peptide Snu66 and Hub-1 protein; Figure S2: Scatter plot of the CABS-dock energy score versus RMSD; Figure S3: Comparison of the ^1^H NMR spectra of the Hub1 protein titrated with the 10-fold excess of peptide-11 and peptide-14; Figure S4: MST analysis of the binding of peptide-11 to Hub1; Figure S5: The NMR experiment of the Hub1 protein titrated with the P4G9 fragment with the pH correction of Hub1; Figure S6: ^1^H NMR spectra of peptide-11 and peptide-14; Table S1: Summary table of interface analysis of PDBePISA; Table S2: Input commands for the docking associated to the peptide from Snu66 (HINDI domain); Table S3: Summary table of the CABS-dock modeling stage; Table S4: Summary table of the CAPRI assessments across all of the modeling stages; Table S5: Summary table of the Fnat values calculated across all of the modeling stages; Table S6: Summary table of the LRMS values; Table S7: Summary RMSD (Å) table of the top 10 medoid in the all-atom representation; Table S8: Structures and code names of the tested fragments. Plate 1; Table S9: Structures and code names of the tested fragments. Plate 2; Table S10: Structures and code names of the tested fragments. Plate 3; Table S11: Structures and code names of the tested fragments. Plate 4; Table S12: Structures and code names of the tested fragments. Plate 5; Table S13: Structures and titration ^1^H and SOFAS HMQC experiments of the initial hits.

## Sample Availability

Samples of the compounds used for fragment-based screening (Tables S8–S12) and peptides 11 and 14 are available from the authors.
