# Correction: Reyes Romero et al. Computer- and NMR-Aided Design of Small-Molecule Inhibitors of the Hub1 Protein. *Molecules* 2022, *27*, 8282

**DOI:** 10.3390/molecules29122926

**Published:** 2024-06-20

**Authors:** Atilio Reyes Romero, Katarzyna Kubica, Radoslaw Kitel, Ismael Rodríguez, Katarzyna Magiera-Mularz, Alexander Dömling, Tad A. Holak, Ewa Surmiak

**Affiliations:** 1 Department of Organic Chemistry, Faculty of Chemistry, Jagiellonian University, Gronostajowa 2, 30-387 Krakow, Poland; 2 Department of Drug Design, University of Groningen, A. Deusinglaan 1, 9713 AV Groningen, The Netherlands

## Change of Affiliations

In the published publication [1], there was an error regarding the affiliation of Alexander Dömling. The affiliation of Professor Dömling with the “Department of Innovative Chemistry, Palackӯ University, CATRIN, Šlechtitelů 241/27, 779 00 Olomouc, Czech Republic” should be removed. Thus, the updated affiliation is as follows: Department of Drug Design, University of Groningen, A. Deusinglaan 1, 9713 AV Groningen, The Netherlands. The reason for the affiliation mix-up is that the coauthor Alexander Dömling changed affiliations from the Netherlands to Czechia during the submission process. The authors state that the scientific conclusions are unaffected. This correction was approved by the Academic Editor. The original publication has also been updated.
