# Supplementary material for: Computer- and NMR-Aided Design of Small-Molecule Inhibitors of the Hub1 Protein
Source: Molecules. 2022 Nov 28;27(23):8282. doi: 10.3390/molecules27238282 (PMC9738620; doi:10.3390/molecules27238282)
Supplement: Supplementary file 1 [file molecules-27-08282-s001.zip › molecules-2026256-supplementary.pdf]

Supplementary Information

For

Computer- and NMR-Aided Design of Small-Molecule Inhibitors of the Hub1 Protein

Atilio Reyes Romero [1,2], Katarzyna Kubica [1], Radoslaw Kitel [1], Ismael Rodríguez [1], Katarzyna Magiera-Mularz [1], Alexander Dömling [2,3], Tad A. Holak [1,*] and Ewa Surmiak [1,*]

[1] Department of Organic Chemistry, Faculty of Chemistry, Jagiellonian University, Gronostajowa 2, 30-387 Krakow, Poland

[2] University of Groningen, Department of Drug Design, A. Deusinglaan 1, 9713 AV Groningen, The Netherlands

[3] Palackȳ University, CATRIN, Department of Innovative Chemistry, Šlechtitelů 241/27 779 00, Olomouc-Holice, Czech Republic

1. SUPPLEMENTARY FIGURES

Figure S1. Close up view of molecular interactions between peptide Snu66 (cyan cartoons) and Hub-1 protein (green surface and cartoons). Van der Waals, hydrogen bonds and ionic contacts are represented as yellow, red, and pink dotted lines respectively.

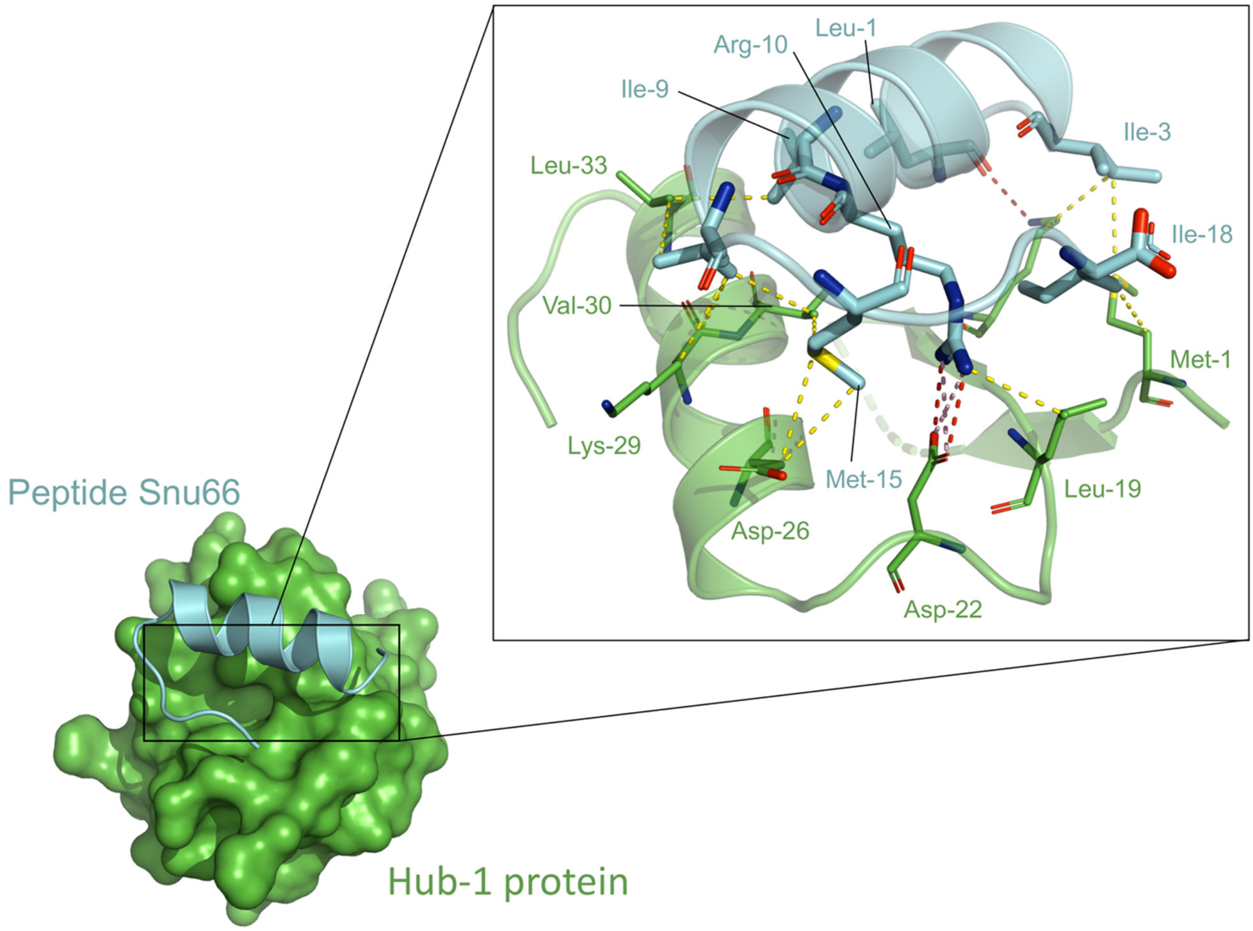

Figure S2. Scatter plot of CABS-dock energy score (y-axis) versus RMSD (x-axis). The RMSD value is calculated only for CA atoms. Blue, red and purples crosses indicate the lowest filtered, lowest medoid and top best models respectively.

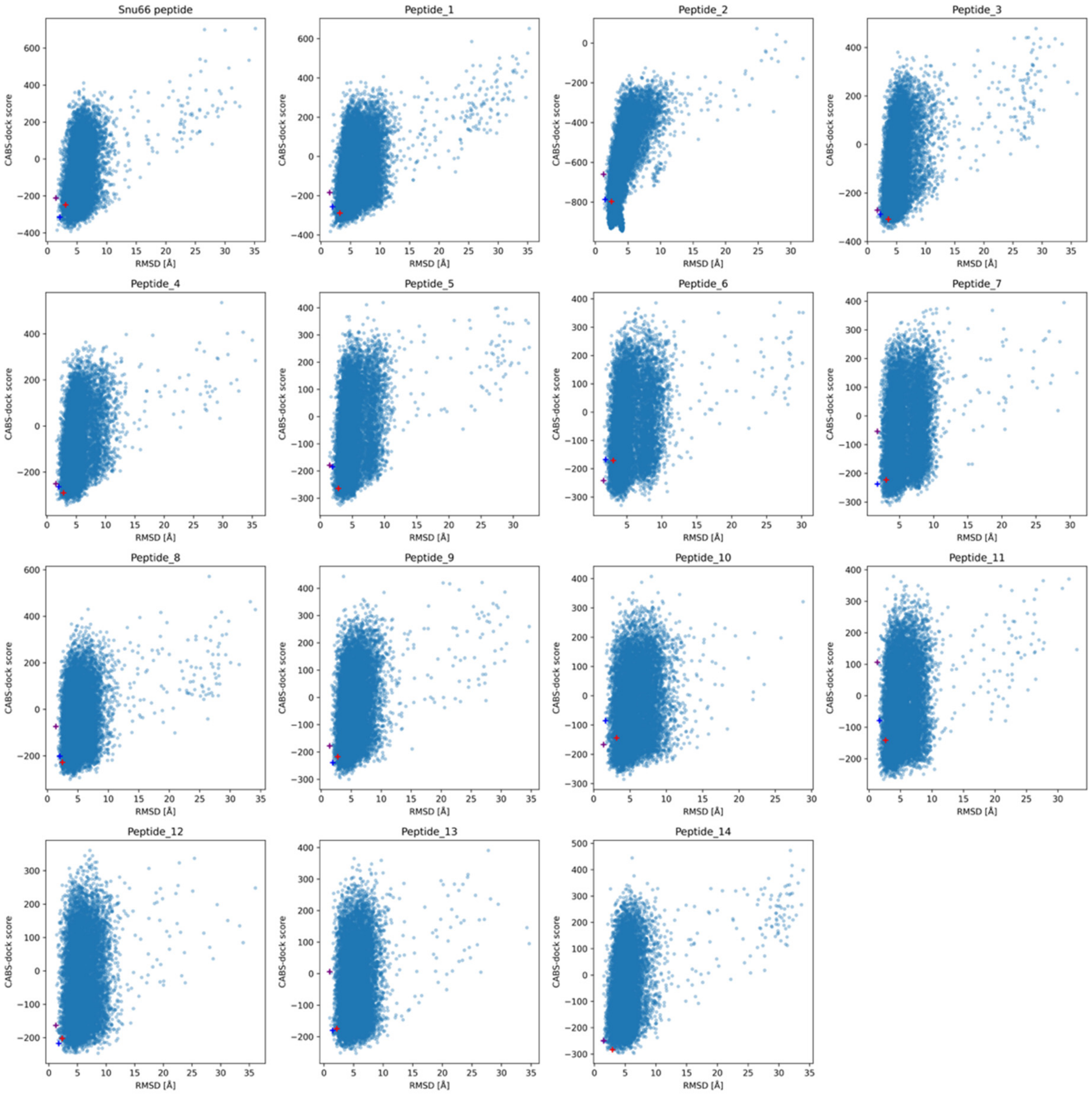

Figure S3. Comparison of the ^{1}H NMR spectra of the Hub1 protein (red) titrated with the 10-fold excess of the peptide-11 (yellow) and peptide-14 (blue). Upper– aliphatic region; Lower – aromatic region

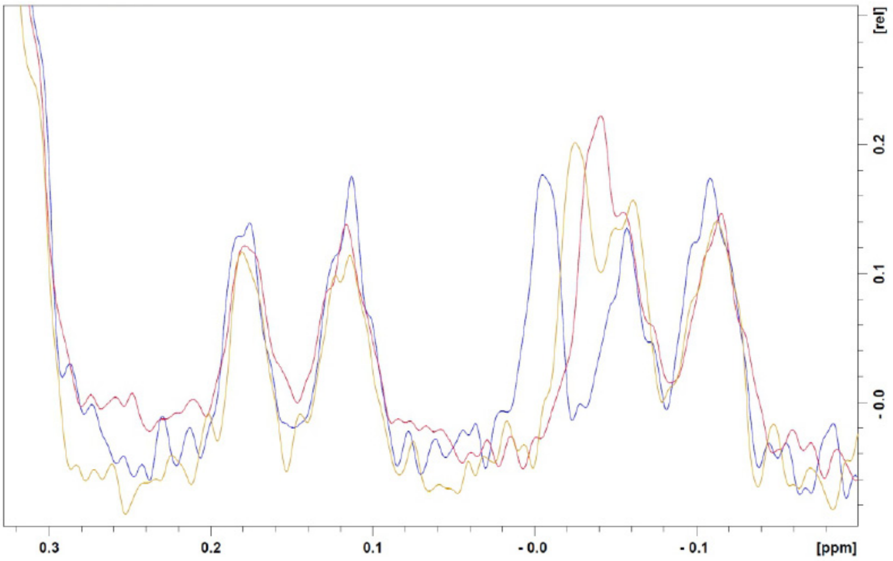

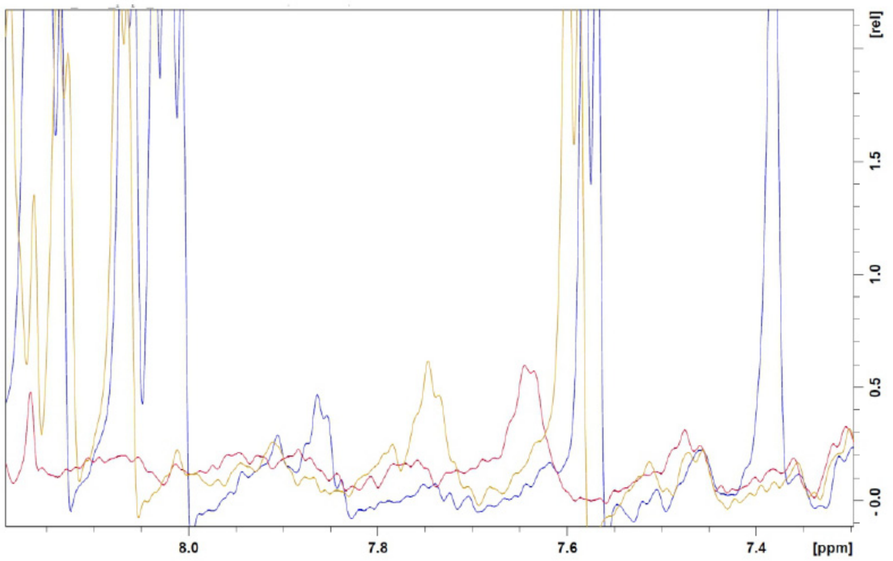

Figure S4. MST analysis of binding **of Peptide-11 to Hub1**

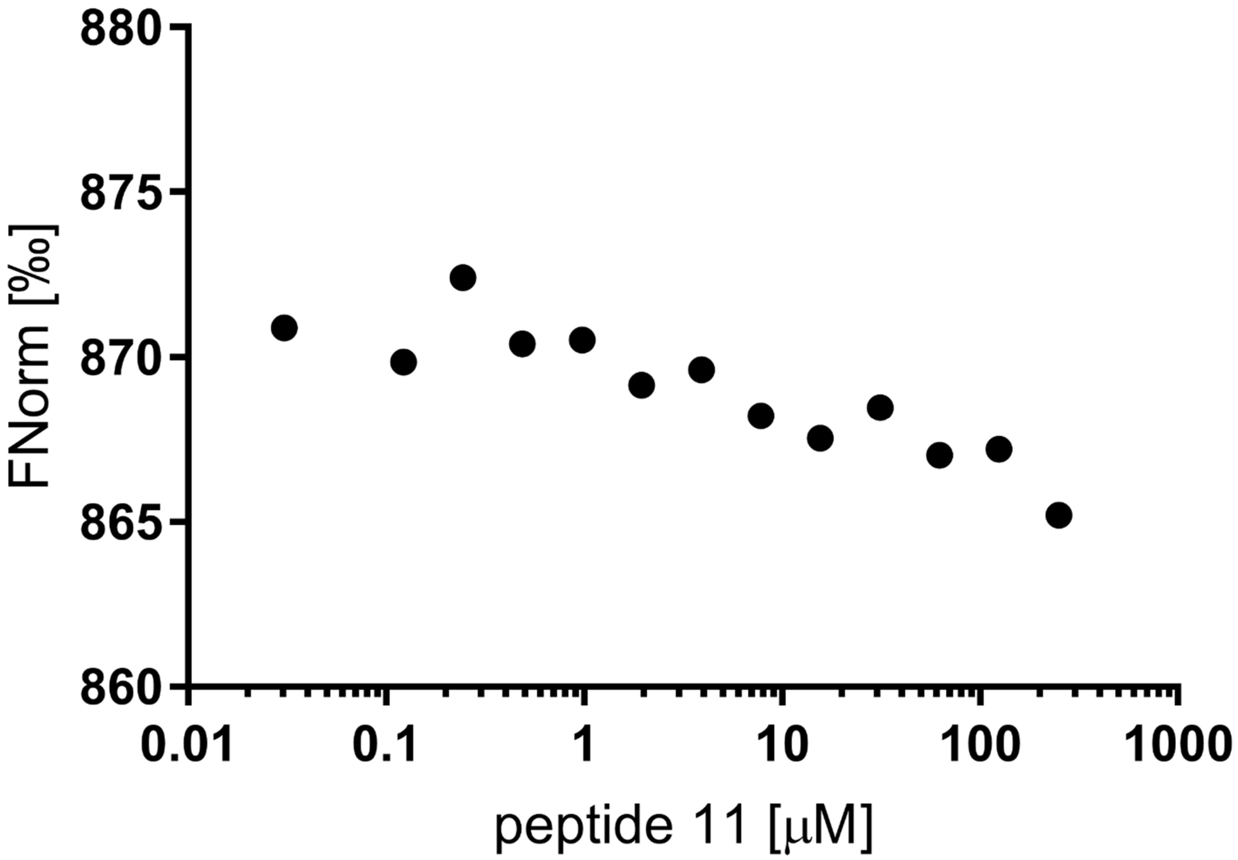

Figure S5. The NMR experiment of Hub1 protein titrated with P4G9 fragment with the pH correction of Hub1. Red – reference Hub1, blue – after 1:10 addition of P4G9, yellow – pH corrected to 6.8, green – pH corrected to 7.2. A. ^{1}H NMR aliphatic region of the spectrum B. ^{1}H NMR aromatic/amide region of the spectrum C. ^{1}H-^{15}N HMQC spectrum

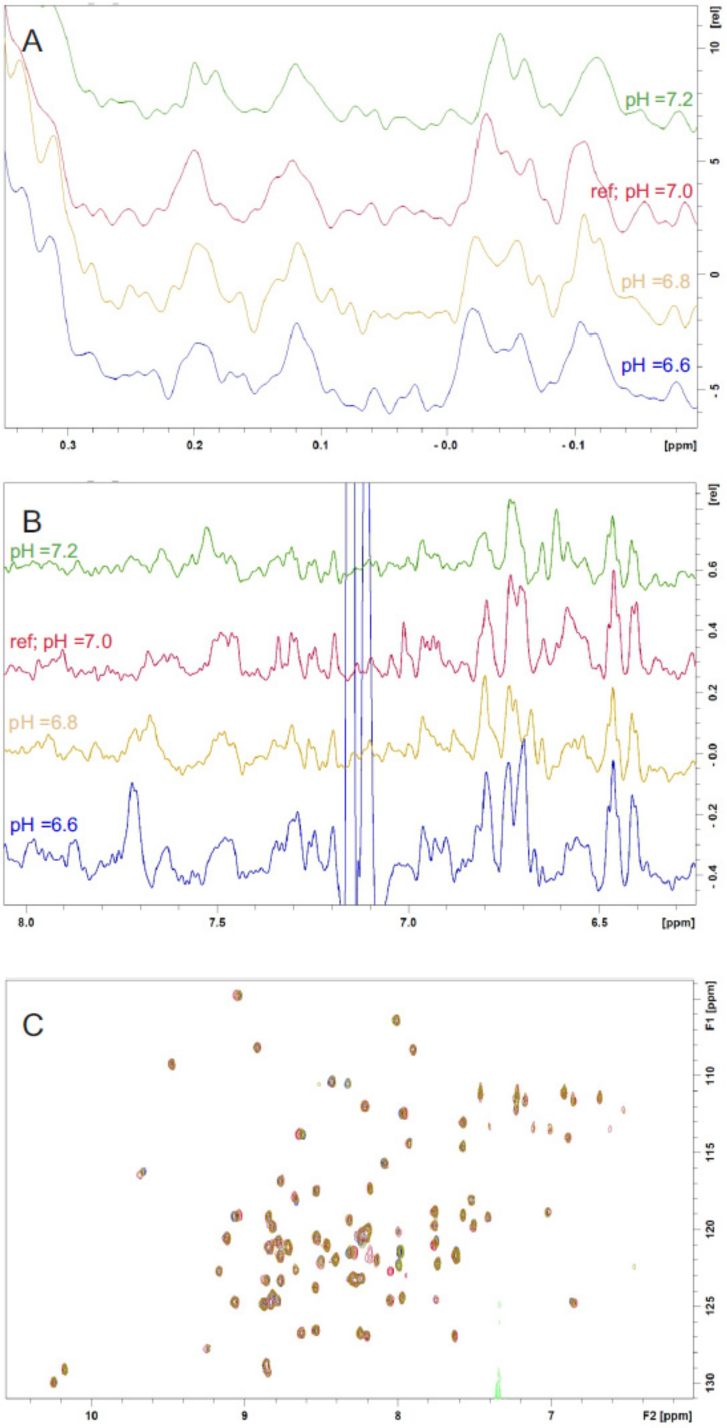

Figure S6. ^{1}H NMR spectra of peptide-11 and peptide-14. Lack of the signal in law aliphatic region and the narrow shape of the peaks indicating that both peptides are lacking secondary structure in the solution. Spectra were measured in H_2O/D_2O with WATERGATE sequence

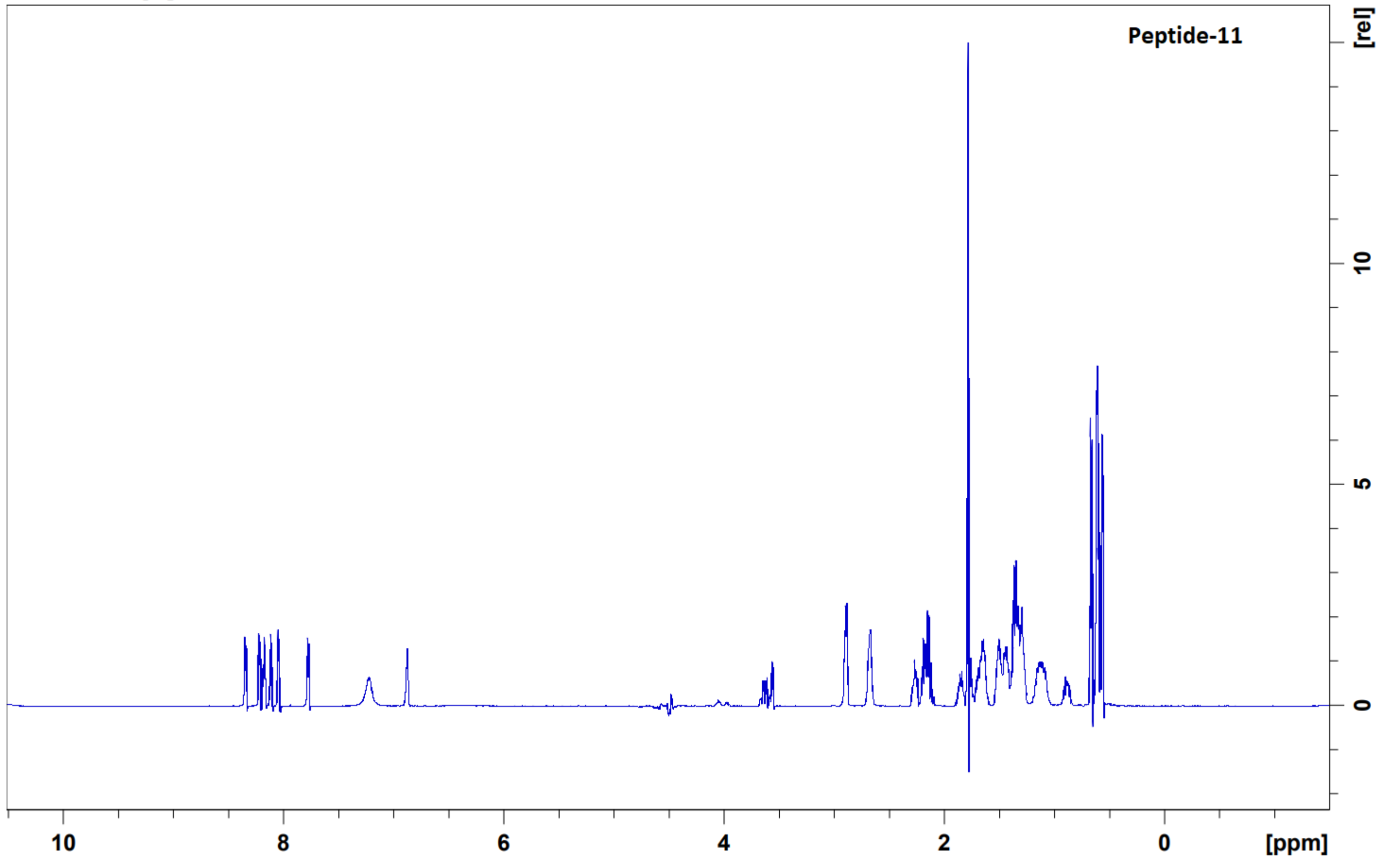

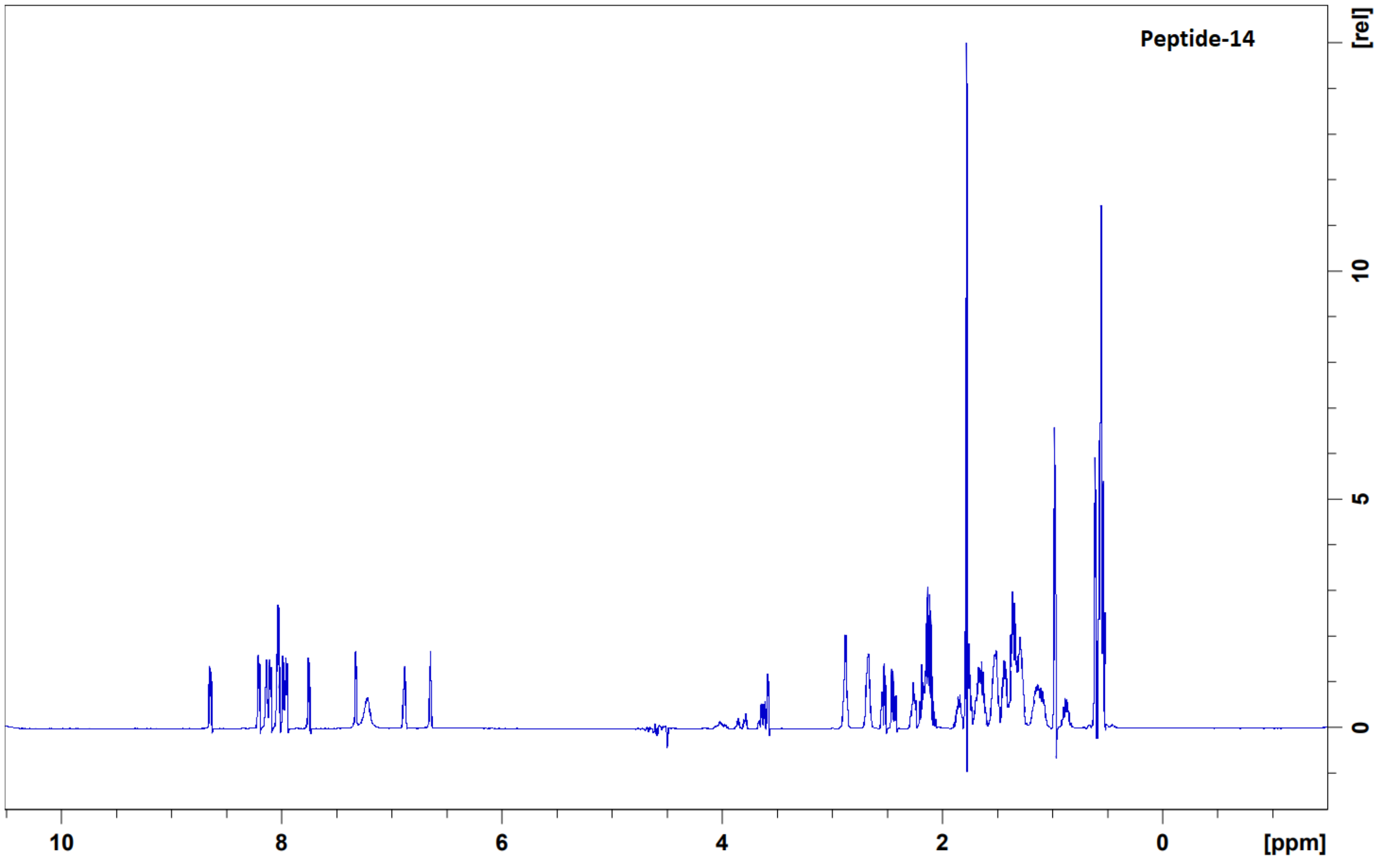

2. SUPPLEMENTAR TABLES

Table S1. Summary table of interface analysis of PDBePISA. The asterisks indicate the amino acids used for the distance restrains used during the molecular docking. $\Delta^{i}G$ = solvatation energy effect, |||: buried area percentage, one bar per 10%, HSDC: Hydrogen/Disulphide bond, Salt bridge or Covalent link, ASA: accessible solvent area, BSA: buried solvent area.

<table>
<tr><th></th><th>Snu-66 peptide</th><th>HSDC</th><th>ASA (Å²)</th><th>BSA (Å²)</th><th>ΔⁱG (kcal/mol)</th></tr>
<tr><td>1</td><td>C: LEU 7</td><td>H</td><td>159.68</td><td>74.70 |||||</td><td>0.12</td></tr>
<tr><td>2</td><td>C: SER 8</td><td></td><td>54.90</td><td>2.34 |</td><td>0.04</td></tr>
<tr><td>3</td><td>C: ILE 9</td><td></td><td>114.45</td><td>40.43 ||||</td><td>0.63</td></tr>
<tr><td>4</td><td>C: GLU 10</td><td></td><td>123.72</td><td>0.00</td><td>0.00</td></tr>
<tr><td>5</td><td>C: GLU 11</td><td></td><td>86.14</td><td>0.00</td><td>0.00</td></tr>
<tr><td>6</td><td>C: THR 12 *</td><td></td><td>37.91</td><td>37.78 ||||||||||</td><td>0.41</td></tr>
<tr><td>7</td><td>C: ASN 13</td><td></td><td>34.65</td><td>0.00</td><td>0.00</td></tr>
<tr><td>8</td><td>C: GLU 14</td><td></td><td>113.00</td><td>0.00</td><td>0.00</td></tr>
<tr><td>9</td><td>C: ILE 15 *</td><td></td><td>89.70</td><td>63.63 ||||||||</td><td>1.01</td></tr>
<tr><td>10</td><td>C: ARG 16 *</td><td>HS</td><td>61.70</td><td>59.23 ||||||||||</td><td>-1.03</td></tr>
<tr><td>11</td><td>C: GLU 17</td><td></td><td>68.55</td><td>0.00</td><td>0.00</td></tr>
<tr><td>12</td><td>C: LYS 18 *</td><td></td><td>92.82</td><td>3.01 |</td><td>0.05</td></tr>
<tr><td>13</td><td>C: LEU 19 *</td><td></td><td>123.10</td><td>68.13 ||||||</td><td>0.95</td></tr>
<tr><td>14</td><td>C: GLY 20</td><td></td><td>71.20</td><td>0.00</td><td>0.00</td></tr>
<tr><td>15</td><td>C: MET 21 *</td><td></td><td>92.29</td><td>63.73 |||||||</td><td>1.48</td></tr>
<tr><td>16</td><td>C: LYS 22</td><td></td><td>69.80</td><td>8.54 ||</td><td>0.14</td></tr>
<tr><td>17</td><td>C:PRO 23</td><td></td><td>103.46</td><td>6.01 |</td><td>-0.07</td></tr>
<tr><td>18</td><td>C: ILE 24 *</td><td></td><td>139.43</td><td>81.55 ||||||</td><td>1.13</td></tr>
</table>

Table S2. Input commands for the docking associated to the peptide from Snu66 (HINDI domain). A detailed description of the command's lines can be found at https://bitbucket.org/lcbio/cabsdock/wiki/Home

Peptide	Commands
Snu66 peptide	CABSdock -M -C -S -i 1M94.pdb -p LSIEETNEIREKLGMKPI:CHHHHHHHHHHCCCCCCC -v4 --dssp-command Users/Atilio/anaconda3/envs/CABSdock/bin/mkdssp --aa-rebuild --reference-pdb 3plu.pdb:A:C --ca-rest-add 3:PEP 18:PEP 7.7 1.0 --ca-rest-add 15:PEP 11:PEP 5.4 1.0 --ca-rest-add 17:PEP 7:PEP 5.6 1.0 --sc-rest-add 17:A 1:PEP 2.8 1 --sc-rest-add 1:A 3:PEP 3.9 1 --sc-rest-add 17:A 3:PEP 3.8 1 --sc-rest-add 17:A 6:PEP 3.3 1 --sc-rest-add 33:A 9:PEP 3.6 1 --sc-rest-add 19:A 10:PEP 3.6 1 --sc-rest-add 22:A 10:PEP 3.9 1 --sc-rest-add 33:A 13:PEP 3.8 1 --sc-rest-add 30:A 13:PEP 3.8 1 --sc-rest-add 29:A 13:PEP 3.9 1 --sc-rest-add 26:A 15:PEP 4.0 1 --sc-rest-add 30:A 15:PEP 3.8 1 --sc-rest-add 1:A 18:PEP 3.4 1
Peptide 1	CABSdock -M -C -S -i 1M94.pdb -p SIEETNEIREKLGMKPI:HHHHHHHHHHCCCCCCC -v4 --dssp-command Users/Atilio/anaconda3/envs/CABSdock/bin/mkdssp --aa-rebuild --reference-pdb 3plu.pdb:A:C --ca-rest-add 2:PEP 17:PEP 7.7 1.0 --ca-rest-add 14:PEP 10:PEP 5.4 1.0 --ca-rest-add 16:PEP 6:PEP 5.6 1.0 --sc-rest-add 1:A 2:PEP 3.9 1.0 --sc-rest-add 17:A 2:PEP 3.8 1 --sc-rest-add 17:A 5:PEP 3.3 1 --sc-rest-add 33:A 8:PEP 3.6 1.0 --sc-rest-add 19:A 9:PEP 3.6 1.0 --sc-rest-add 22:A 9:PEP 3.9 1.0 --sc-rest-add 33:A 12:PEP 3.8 1.0 --sc-rest-add 30:A 12:PEP 3.8 1.0 --sc-rest-add 29:A 12:PEP 3.9 1.0 --sc-rest-add 26:A 14:PEP 4.0 1.0 --sc-rest-add 30:A 14:PEP 3.8 1 --sc-rest-add 1:A 17:PEP 3.4 1.0
Peptide 2	CABSdock -M -C -S -i 1M94.pdb -p IEETNEIREKLGMKPI:HHHHHHHHHCCCCCCC -v4 --dssp-command Users/Atilio/anaconda3/envs/CABSdock/bin/mkdssp --aa-rebuild --reference-pdb 3plu.pdb:A:C --ca-rest-add 1:PEP 16:PEP 7.7 1.0 --ca-rest-add 9:PEP 13:PEP 5.4 1.0 --ca-rest-add 15:PEP 5:PEP 5.6 1.0 --sc-rest-add 17:A 1:PEP 3.8 1 --sc-rest-add 17:A 4:PEP 3.3 1 --sc-rest-add 33:A 7:PEP 3.6 1 --sc-rest-add 19:A 8:PEP 3.6 1 --sc-rest-add 22:A 8:PEP 3.9 1 --sc-rest-add 33:A 11:PEP 3.8 1 --sc-rest-add 30:A 11:PEP 3.8 1 --sc-rest-add 29:A 11:PEP 3.9 1 --sc-rest-add 26:A 13:PEP 4.0 1 --sc-rest-add 30:A 13:PEP 3.8 1 --sc-rest-add 1:A 16:PEP 3.4 1
Peptide 3	CABSdock -M -C -S -i 1M94.pdb -p EETNEIREKLGMKPI:HHHHHHHHCCCCCCC -v4 --dssp-command Users/Atilio/anaconda3/envs/CABSdock/bin/mkdssp --aa-rebuild --reference-pdb 3plu.pdb:A:C --ca-rest-add 12:PEP 8:PEP 5.4 1.0 --ca-rest-add 14:PEP 4:PEP 5.6 1.0 --sc-rest-add 17:A 3:PEP 3.3 1 --sc-rest-add 33:A 6:PEP 3.6 1 --sc-rest-add 19:A 7:PEP 3.6 1 --sc-rest-add 22:A 7:PEP 3.9 1 --sc-rest-add 33:A 10:PEP 3.8 1 --sc-rest-add 30:A 10:PEP 3.8 1 --sc-rest-add 29:A 10:PEP 3.9 1 --sc-rest-add 26:A 12:PEP 4.0 1 --sc-rest-add 30:A 12:PEP 3.8 1 --sc-rest-add 1:A 15:PEP 3.4 1
Peptide 4	CABSdock -M -C -S -i 1M94.pdb -p ETNEIREKLGMKPI:HHHHHHHCCCCCCC -v4 --dssp-command Users/Atilio/anaconda3/envs/CABSdock/bin/mkdssp --aa-rebuild --reference-pdb 3plu.pdb:A:C --ca-rest-add 11:PEP 7:PEP 5.4 1.0 --ca-rest-add 13:PEP 3:PEP 5.6 1.0 --sc-rest-add 17:A 2:PEP 3.3 1 --sc-rest-add 33:A 5:PEP 3.6 1 --sc-rest-add 19:A 6:PEP 3.6 1 --sc-rest-add 22:A 6:PEP 3.9 1 --sc-rest-add 33:A 9:PEP 3.8 1 --sc-rest-add 30:A 9:PEP 3.8 1 --sc-rest-add 29:A 9:PEP 3.9 1 --sc-rest-add 26:A 11:PEP 4.0 1 --sc-rest-add 30:A 11:PEP 3.8 1 --sc-rest-add 1:A 14:PEP 3.4 1

Peptide 5	CABSdock -M -C -S -i 1M94.pdb -p TNEIREKLGMKPI:HHHHHHCCCCCCC -v4 --dssp-command Users/Atilio/anaconda3/envs/CABSdock/bin/mkdssp --aa-rebuild --reference-pdb 3plu.pdb:A:C 1.0 --ca-rest-add 10:PEP 6:PEP 5.4 1.0 --ca-rest-add 12:PEP 2:PEP 5.6 1.0 --sc-rest-add 17:A 1:PEP 3.3 1 --sc-rest-add 33:A 4:PEP 3.6 1 --sc-rest-add 19:A 5:PEP 3.6 1 --sc-rest-add 22:A 5:PEP 3.9 1 --sc-rest-add 33:A 8:PEP 3.8 1 --sc-rest-add 30:A 8:PEP 3.8 1 --sc-rest-add 29:A 8:PEP 3.9 1 --sc-rest-add 26:A 10:PEP 4.0 1 --sc-rest-add 30:A 10:PEP 3.8 1 --sc-rest-add 1:A 13:PEP 3.4 1
Peptide 6	CABSdock -M -C -S -i 1M94.pdb -p NEIREKLGMKPI:HHHHHCCCCCCC -v4 --dssp-command Users/Atilio/anaconda3/envs/CABSdock/bin/mkdssp --aa-rebuild --reference-pdb 3plu.pdb:A:C --ca-rest-add 9:PEP 5:PEP 5.4 1.0 --ca-rest-add 11:PEP 1:PEP 5.6 1.0 --sc-rest-add 33:A 3:PEP 3.6 1 --sc-rest-add 19:A 4:PEP 3.6 1 --sc-rest-add 22:A 4:PEP 3.9 1 --sc-rest-add 33:A 7:PEP 3.8 1 --sc-rest-add 30:A 7:PEP 3.8 1 --sc-rest-add 29:A 7:PEP 3.9 1 --sc-rest-add 26:A 9:PEP 4.0 1 --sc-rest-add 30:A 9:PEP 3.8 1 --sc-rest-add 1:A 12:PEP 3.4 1
Peptide 7	CABSdock -M -C -S -i 1M94.pdb -p EIREKLGMKPI:HHHHCCCCCCC -v4 --dssp-command Users/Atilio/anaconda3/envs/CABSdock/bin/mkdssp --aa-rebuild --reference-pdb 3plu.pdb:A:C --ca-rest-add 3:PEP 10:PEP 6.3 1.0 --ca-rest-add 4:PEP 8:PEP 5.4 1.0 --ca-rest-add 7:PEP 5:PEP 5.4 1.0 --sc-rest-add 33:A 2:PEP 3.6 1 --sc-rest-add 19:A 3:PEP 3.6 1 --sc-rest-add 22:A 3:PEP 3.9 1 --sc-rest-add 33:A 6:PEP 3.8 1 --sc-rest-add 30:A 6:PEP 3.8 1 --sc-rest-add 29:A 6:PEP 3.9 1 --sc-rest-add 26:A 8:PEP 4.0 1 --sc-rest-add 30:A 8:PEP 3.8 1 --sc-rest-add 1:A 11:PEP 3.4 1
Peptide 8	CABSdock -M -C -S -i 1M94.pdb -p IREKLGMKPI:HHHCCCCCCC -v4 --dssp-command Users/Atilio/anaconda3/envs/CABSdock/bin/mkdssp --aa-rebuild --reference-pdb 3plu.pdb:A:C --ca-rest-add 2:PEP 9:PEP 6.3 1.0 --ca-rest-add 3:PEP 7:PEP 5.4 1.0 --ca-rest-add 6:PEP 4:PEP 5.4 1.0 --sc-rest-add 33:A 1:PEP 3.6 1 --sc-rest-add 19:A 2:PEP 3.6 1 --sc-rest-add 22:A 2:PEP 3.9 1 --sc-rest-add 33:A 5:PEP 3.8 1 --sc-rest-add 30:A 5:PEP 3.8 1 --sc-rest-add 29:A 5:PEP 3.9 1 --sc-rest-add 26:A 7:PEP 4.0 1 --sc-rest-add 30:A 7:PEP 3.8 1 --sc-rest-add 1:A 10:PEP 3.4 1
Peptide 9	CABSdock -M -C -S -i 1M94.pdb -p IREKLGMKP:HHHCCCCCC -v4 --dssp-command Users/Atilio/anaconda3/envs/CABSdock/bin/mkdssp --aa-rebuild --reference-pdb 3plu.pdb:A:C --ca-rest-add 2:PEP 9:PEP 6.3 1.0 --ca-rest-add 3:PEP 7:PEP 5.4 1.0 --ca-rest-add 6:PEP 4:PEP 5.4 1.0 --sc-rest-add 33:A 1:PEP 3.6 1 --sc-rest-add 19:A 2:PEP 3.6 1 --sc-rest-add 22:A 2:PEP 3.9 1 --sc-rest-add 33:A 5:PEP 3.8 1 --sc-rest-add 30:A 5:PEP 3.8 1 --sc-rest-add 29:A 5:PEP 3.9 1 --sc-rest-add 26:A 7:PEP 4.0 1 --sc-rest-add 30:A 7:PEP 3.8 1
Peptide 10	CABSdock -M -C -S -i 1M94.pdb -p IREKLGMK:HHHCCCCC -v4 --dssp-command Users/Atilio/anaconda3/envs/CABSdock/bin/mkdssp --aa-rebuild --reference-pdb 3plu.pdb:A:C --ca-rest-add 4:PEP 4:PEP 5.4 1.0 --ca-rest-add 3:PEP 7:PEP 5.4 1.0 --sc-rest-add 33:A 1:PEP 3.6 1 --sc-rest-add 19:A 2:PEP 3.6 1 --sc-rest-add 22:A 2:PEP 3.9 1 --sc-rest-add 33:A 5:PEP 3.8 1 --sc-rest-add 30:A 5:PEP 3.8 1 --sc-rest-add 29:A 5:PEP 3.9 1 --sc-rest-add 26:A 7:PEP 4.0 1 --sc-rest-add 30:A 7:PEP 3.8 1
Peptide 11	CABSdock -M -C -S -i 1M94.pdb -p IREKLGM:HHHCCCC -v4 --dssp-command Users/Atilio/anaconda3/envs/CABSdock/bin/mkdssp --aa-rebuild --reference-pdb 3plu.pdb:A:C --ca-rest-add 4:PEP 4:PEP 5.4 1.0 --ca-rest-add 3:PEP 7:PEP 5.4 1.0 --sc-rest-add 33:A 1:PEP 3.6 1 --sc-rest-add 19:A 2:PEP 3.6 1 --sc-rest-add 22:A 2:PEP 3.9 1 --sc-rest-add 33:A 5:PEP 3.8 1 --sc-rest-add 30:A 5:PEP 3.8 1 --sc-rest-add 29:A 5:PEP 3.9 1 --sc-rest-add 26:A 7:PEP 4.0 1 --sc-rest-add 30:A 7:PEP 3.8 1
Peptide 12	CABSdock -M -C -S -i 1M94.pdb -p IREKLG:HHHCCC -v4 --dssp-command Users/Atilio/anaconda3/envs/CABSdock/bin/mkdssp --aa-rebuild --reference-pdb 3plu.pdb:A:C --ca-rest-add 6:PEP 4:PEP 5.4 1.0 --sc-rest-add 33:A 1:PEP 3.6 1 --sc-rest-add 19:A 2:PEP 3.6 1 --sc-rest-add 22:A 2:PEP 3.9 1 --sc-rest-add 33:A 5:PEP 3.8 1 --sc-rest-add 30:A 5:PEP 3.8 1 --sc-rest-add 29:A 5:PEP 3.9 1

Peptide 13	CABSdock -M -C -S -i 1M94.pdb -p IREKL:HHHCC -v4 --dssp-command Users/Atilio/anaconda3/envs/CABSdock/bin/mkdssp --aa-rebuild --reference-pdb 3plu.pdb:A:C --sc-rest-add 33:A 1:PEP 3.6 1 --sc-rest-add 19:A 2:PEP 3.6 1 --sc-rest-add 22:A 2:PEP 3.9 1 --sc-rest-add 33:A 5:PEP 3.8 1 --sc-rest-add 30:A 5:PEP 3.8 1 --sc-rest-add 29:A 5:PEP 3.9 1
Peptide 14	CABSdock -M -C -S -i 1M94.pdb -p TNEIREKLGM:HHHHHHCCCC -v4 --dssp-command Users/Atilio/anaconda3/envs/CABSdock/bin/mkdssp --aa-rebuild --reference-pdb 3plu.pdb:A:C --ca-rest-add 6:PEP 10:PEP 5.4 1.0 --sc-rest-add 17:A 1:PEP 3.3 1 --sc-rest-add 33:A 4:PEP 3.6 1 --sc-rest-add 19:A 5:PEP 3.6 1 --sc-rest-add 22:A 5:PEP 3.9 1 --sc-rest-add 33:A 8:PEP 3.8 1 --sc-rest-add 30:A 8:PEP 3.8 1 --sc-rest-add 29:A 8:PEP 3.9 1 --sc-rest-add 26:A 10:PEP 4.0 1 --sc-rest-add 30:A 10:PEP 3.8 1

Table S3. Summary table of CABS-dock modeling stage.

ID	Lowest				Filtered				Medoid			
	Replica	ID	RMSD (Å)	CABS-dock score (kcal/mol)	Replica	ID	RMSD (Å)	CABS-dock score (kcal/mol	Replica	ID	RMSD (Å)	CABS-dock score (kcal/mol
Peptide_1	3	913	1.47	-183.75	0	224	1.96	-255.68	8	996	3.19	-288.23
Peptide_2	3	413	1.24	-659.76	1	710	1.48	-786.36	9	874	2.46	-796.55
Peptide_3	6	906	1.68	-270.68	1	636	2.20	-287.51	2	904	3.57	-307.80
Peptide_4	5	50	1.51	-250.34	6	948	1.98	-263.37	8	693	2.80	-290.10
Peptide_5	4	276	1.47	-178.87	8	901	1.90	-184.32	4	52	2.82	-264.19
Peptide_6	9	1000	1.64	-241.73	4	395	1.94	-167.49	1	660	3.03	-171.15
Peptide_7	3	159	1.74	-53.89	8	823	1.76	-237.53	8	976	3.04	-222.90
Peptide_8	0	90	1.38	-73.08	6	807	2.02	-201.25	8	617	2.45	-227.45
Peptide_9	8	361	1.41	-177.39	0	265	1.92	-238.99	8	735	2.75	-217.15
Peptide_10	1	981	1.36	-166.54	7	989	1.63	-85.14	2	668	3.15	-144.10
Peptide_11	8	184	1.32	106.29	5	428	1.66	-78.75	2	429	2.63	-140.98
Peptide_12	6	826	1.23	-163.32	3	906	1.72	-217.14	8	643	2.33	-202.14
Peptide_13	1	475	0.97	6.22	6	947	1.45	-179.74	4	767	2.16	-174.60
Peptide_14	3	11	1.48	-248.99	0	891	1.49	-249.93	7	859	2.93	-283.39
Snu66 peptide	6	646	1.41	-210.99	4	293	2.07	-314.78	8	123	3.06	-247.89

Table S4. Summary table of CAPRI assessments across all the modeling stages.

CAPRI		CA representation			AA representation		
		CABS - dock simulation			ca2all.py		
**ID**	Length	Medoid	Filtered	Lowest	Medoid	Filtered	Lowest
Peptide-1	17	Incorrect	Incorrect	Incorrect	Acceptable	Medium	Medium
Peptide-2	16	Incorrect	Incorrect	Incorrect	Medium	Medium	Medium
Peptide-3	15	Incorrect	Incorrect	Incorrect	Acceptable	Medium	Medium
Peptide-4	14	Incorrect	Incorrect	Incorrect	Acceptable	Medium	Medium
Peptide-5	13	Incorrect	Incorrect	Incorrect	Acceptable	Medium	Acceptable
Peptide-6	12	Incorrect	Incorrect	Incorrect	Acceptable	Medium	Medium
Peptide-7	11	Incorrect	Incorrect	Incorrect	Acceptable	Medium	Medium
Peptide-8	10	Incorrect	Incorrect	Incorrect	Acceptable	Medium	Acceptable
Peptide-9	9	Incorrect	Incorrect	Incorrect	Acceptable	Medium	Medium
Peptide-10	8	Incorrect	Incorrect	Incorrect	Acceptable	Medium	Medium
Peptide-11	7	Incorrect	Incorrect	Incorrect	Acceptable	Medium	Acceptable
Peptide-12	6	Incorrect	Incorrect	Incorrect	Acceptable	Medium	Medium
Peptide-13	5	Incorrect	Incorrect	Incorrect	Acceptable	Medium	Medium
Peptide-14	10	Incorrect	Incorrect	Incorrect	Acceptable	Medium	Acceptable
Snu-66 peptide	18	Incorrect	Incorrect	Incorrect	Acceptable	Medium	Medium

Table S5. Summary table of Fnat values calculated across all the modeling stages. Fnat value computation is deprecated in models in form of CA representation.

**Fnat**		**CA representation**			**AA representation**		
		CABS - dock simulation			ca2all.py		
**ID**	Length	Medoid	Filtered	Lowest	Medoid	Filtered	Lowest
Peptide-1	17	0	0	0	0.88	0.56	0.63
Peptide-2	16	0	0	0	0.75	0.56	0.69
Peptide-3	15	0	0	0	0.79	0.57	0.79
Peptide-4	14	0	0	0	0.79	0.64	0.64
Peptide-5	13	0	0	0	0.64	0.71	0.43
Peptide-6	12	0	0	0	0.69	0.69	0.77
Peptide-7	11	0	0	0	0.69	0.54	0.54
Peptide-8	10	0	0	0	0.77	0.62	0.46
Peptide-9	9	0	0	0	0.90	0.80	0.60
Peptide-10	8	0	0	0	0.80	0.80	0.70
Peptide-11	7	0	0	0	0.90	0.60	0.40
Peptide-12	6	0	0	0	0.43	0.71	0.57
Peptide-13	5	0	0	0	0.57	0.57	0.71
Peptide-14	10	0	0	0	0.46	0.64	0.46
Snu-66 peptide	18	0	0	0	0.72	0.72	0.56

Table S6. Summary table of LRMS values.

LRMS (Å)		CA representation<br>CABS - dock simulation			AA representation<br>ca2all.py		
**ID**	Length	Medoid	Filtered	Lowest	Medoid	Filtered	Lowest
Peptide-1	17	3.19	1.96	1.48	3.06	1.70	1.43
Peptide-2	16	2.49	1.45	1.29	2.33	1.18	1.04
Peptide-3	15	3.55	2.18	1.73	3.33	1.94	1.43
Peptide-4	14	2.85	1.94	1.52	2.55	1.84	1.31
Peptide-5	13	2.88	1.98	1.61	2.73	1.66	1.79
Peptide-6	12	3.09	1.99	1.69	3.02	1.96	1.48
Peptide-7	11	3.05	1.81	1.79	3.00	1.89	1.55
Peptide-8	10	2.53	2.01	1.48	2.33	1.99	1.37
Peptide-9	9	2.82	1.90	1.50	2.82	1.85	1.68
Peptide-10	8	3.21	1.61	1.37	3.23	1.70	1.46
Peptide-11	7	2.65	1.75	1.29	2.56	1.79	1.37
Peptide-12	6	2.37	1.82	1.27	2.74	2.07	1.09
Peptide-13	5	2.20	1.37	0.88	2.46	1.71	1.23
Peptide-14	10	2.99	1.51	1.38	2.96	1.66	1.48
Snu-66 peptide	18	3.17	2.20	1.40	2.90	1.97	1.20

Table S7. Summary RMSD (Å) table of top 10 medoid in all atom representation.

ID	top_1	top_2	top_3	top_4	top_5	top_6	top_7	top_8	top_9	top_10
Snu66 peptide	5.09	4.89	3.79	4.30	3.06	3.46	5.50	6.65	5.62	6.42
Peptide 1	3.29	4.90	3.19	4.92	5.68	5.53	5.31	7.93	6.10	7.34
Peptide 2	2.25	4.63	4.44	4.96	2.81	5.17	4.18	6.27	5.24	3.72
Peptide 3	3.58	3.57	4.51	5.29	5.29	4.49	4.98	4.07	5.37	5.26
Peptide 4	4.62	4.63	3.80	4.92	3.63	5.59	2.80	3.97	3.17	4.32
Peptide 5	2.85	3.83	4.93	4.35	2.82	5.40	3.87	3.49	4.69	4.58
Peptide 6	4.50	3.82	3.03	3.81	3.74	4.75	3.61	3.46	4.08	5.14
Peptide 7	3.09	4.29	3.78	4.23	3.83	4.67	3.04	3.83	4.73	5.65
Peptide 8	3.93	3.00	3.51	4.04	4.34	3.52	3.50	2.45	4.64	3.32
Peptide 9	3.64	4.81	2.83	2.75	4.06	4.25	4.52	3.78	3.85	4.32
Peptide 10	3.67	3.15	4.52	3.29	3.33	3.46	4.90	3.96	4.75	5.08
Peptide 11	3.84	4.59	3.22	3.00	2.63	3.52	2.69	3.40	2.85	5.32
Peptide 12	4.06	2.33	3.83	4.82	4.11	8.10	4.33	4.66	5.31	3.46
Peptide 13	2.81	4.22	3.70	4.22	3.64	4.88	3.09	2.71	2.16	6.86
Peptide 14	5.01	4.62	3.62	4.26	2.93	5.12	6.35	5.38	5.09	5.94

Table S8. Structures and code names of the tested fragments. Plate 1

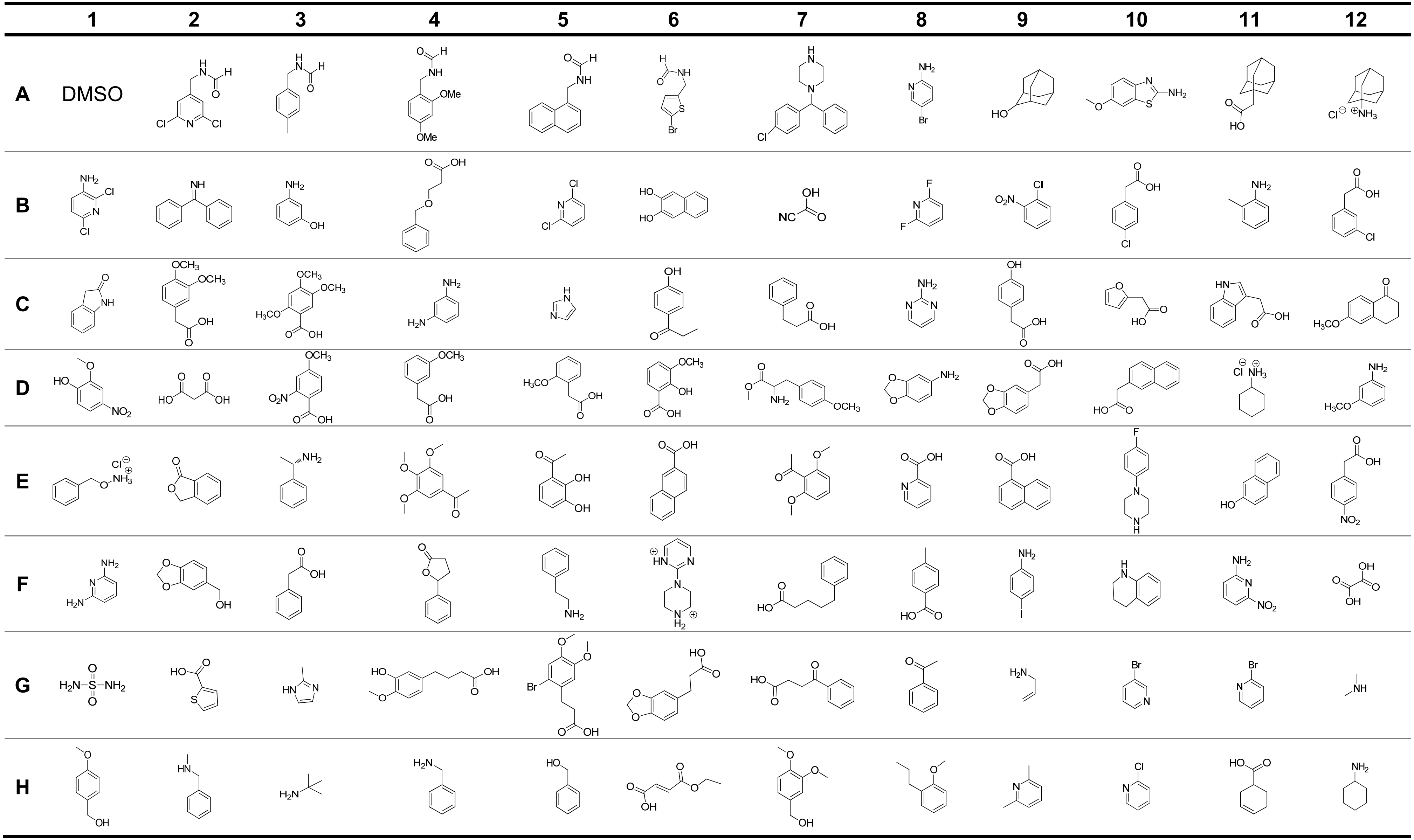

Table S9. Structures and code names of the tested fragments. Plate 2

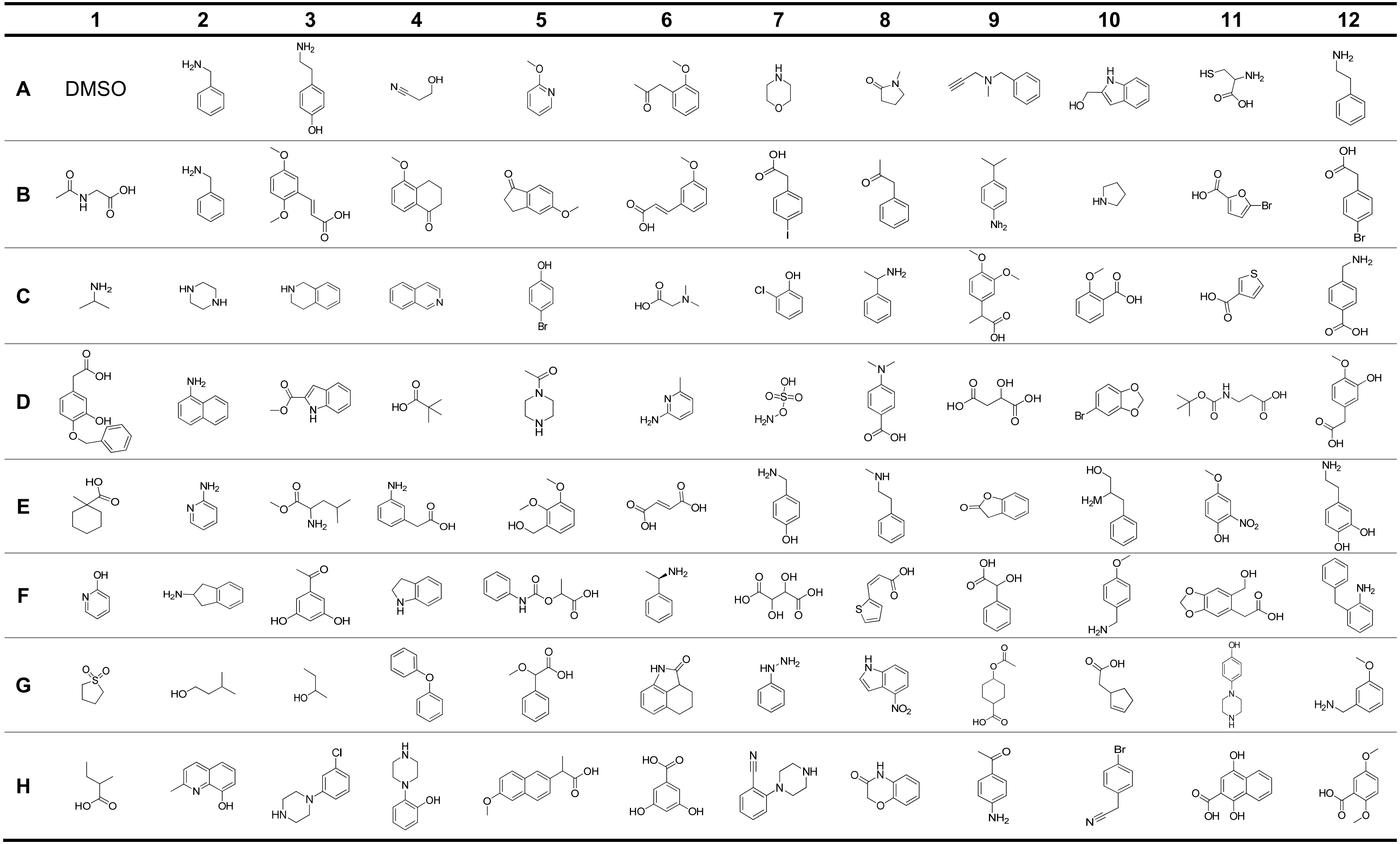

Table S10. Structures and code names of the tested fragments. Plate 3

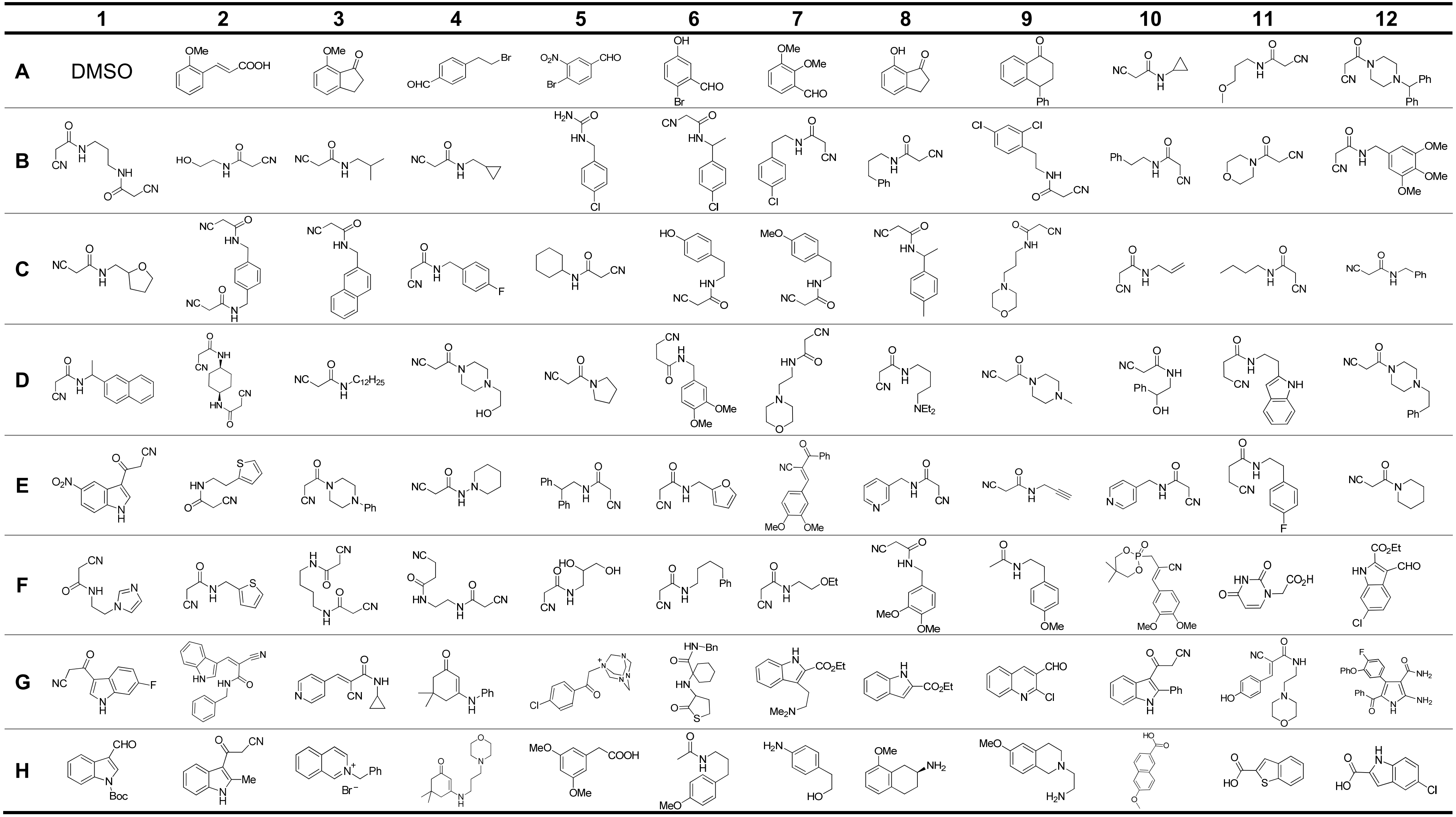

Table S11. Structures and code names of the tested fragments. Plate 4

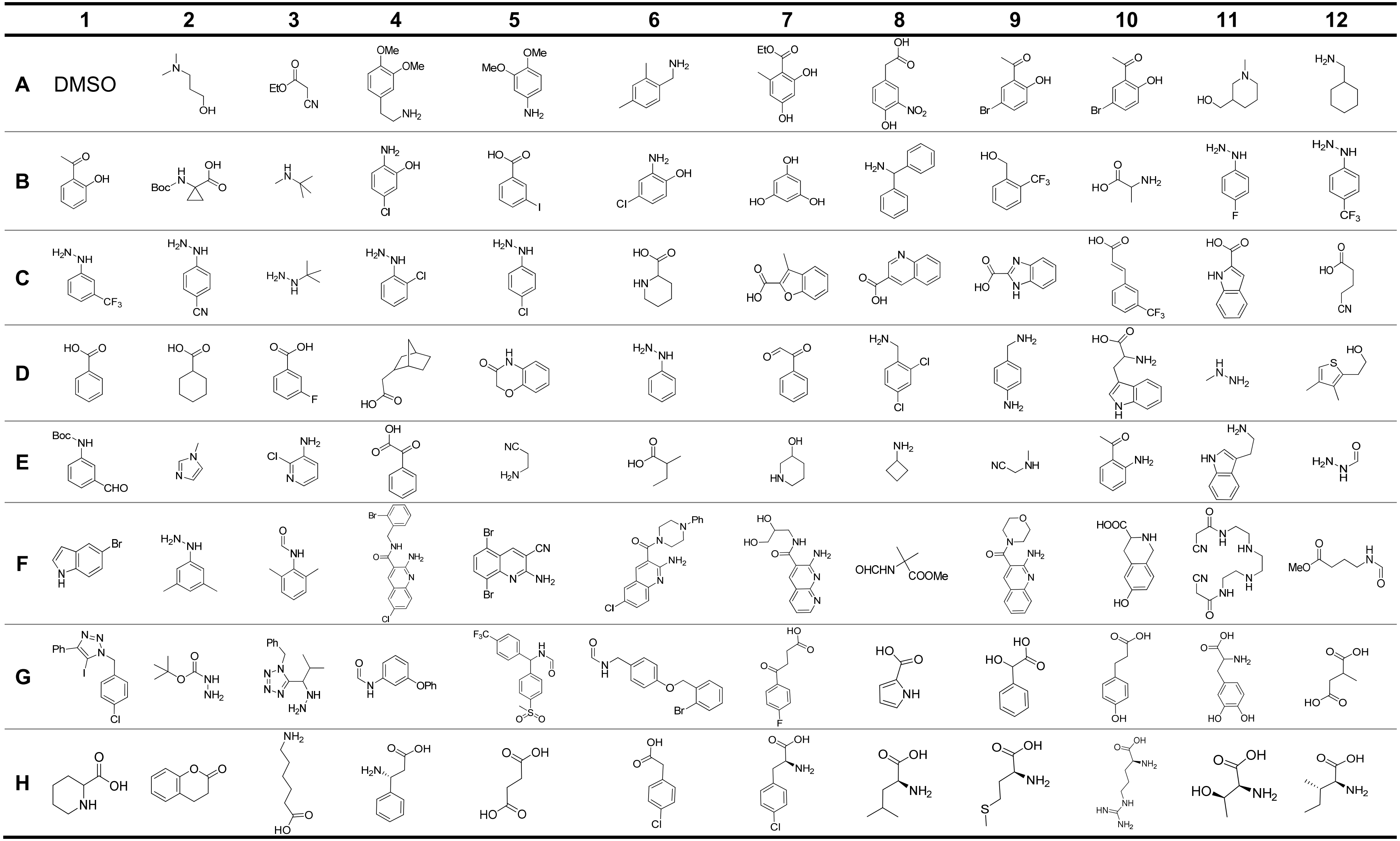

Table S12. Structures and code names of the tested fragments. Plate 5

Columns: 1 2 3 4 5 6 7 8 9 10 11 12

A DMSO (A1)

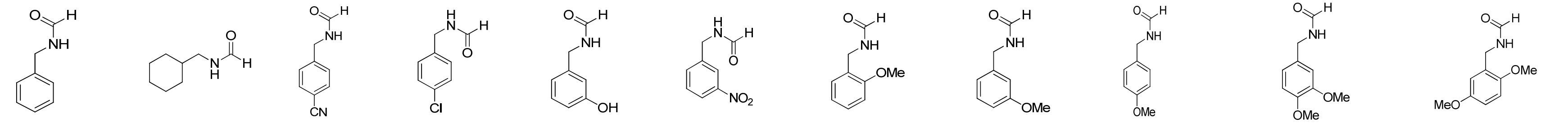

B

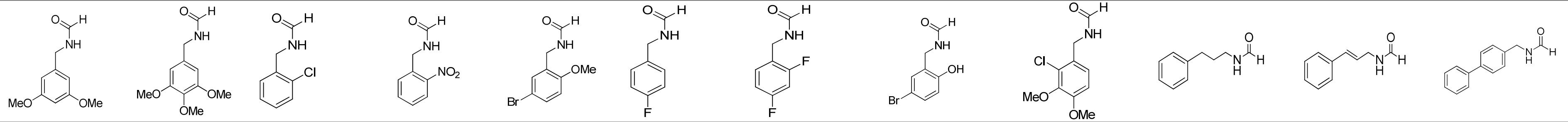

C

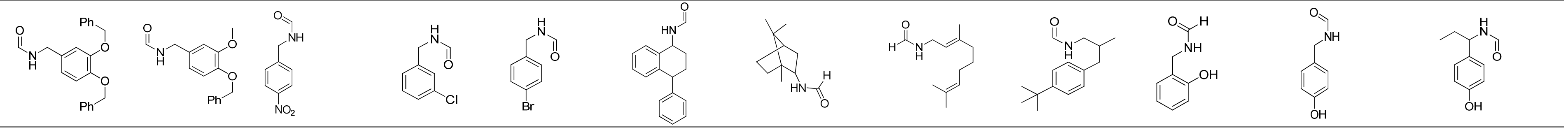

D

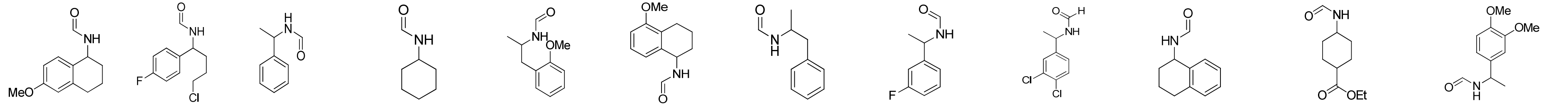

E

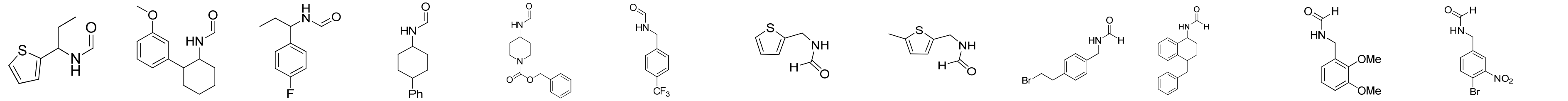

F

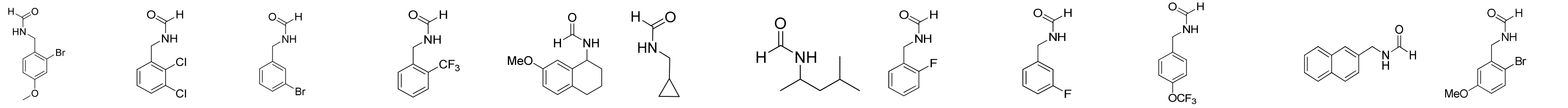

G

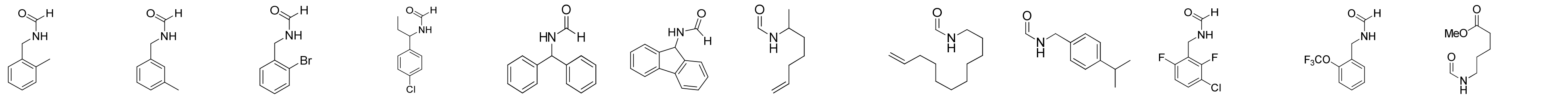

H

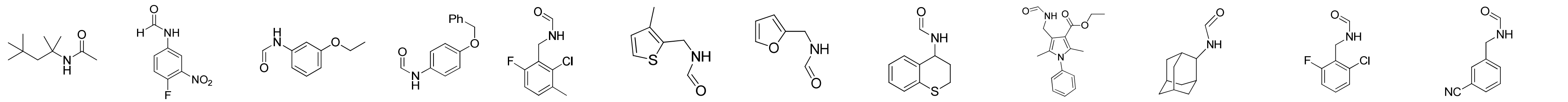

Table S13. Structures and titration ^{1}H and SOFAS HMQC experiments of the initial hits. Red – reference Hub1 titrated with equal amount of DMSO, Blue - Hub1: ligand ratio 1:10

Name	Structure	^{1}H spectrum	HMQC spectrum
P1C3	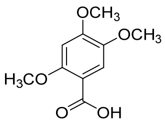	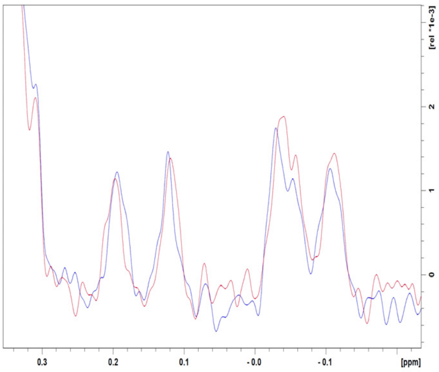	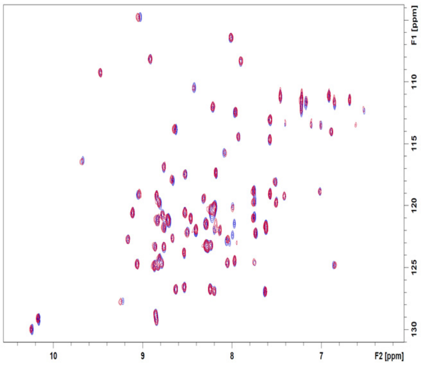
P1C11	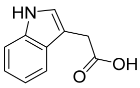	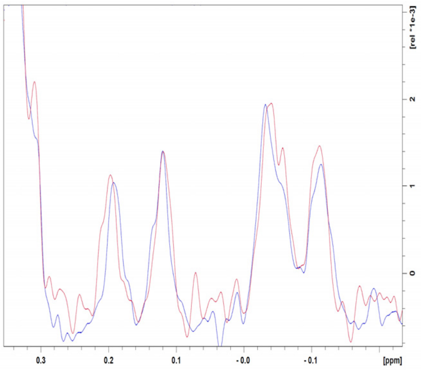	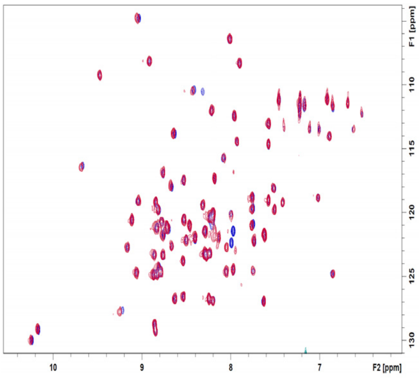
P1D2	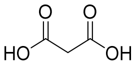	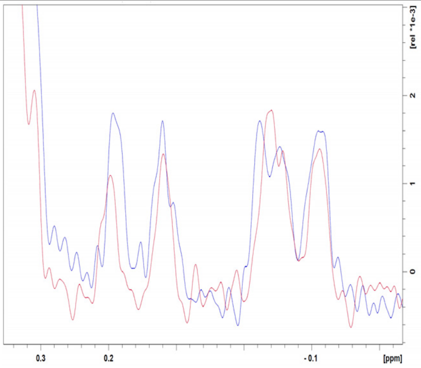	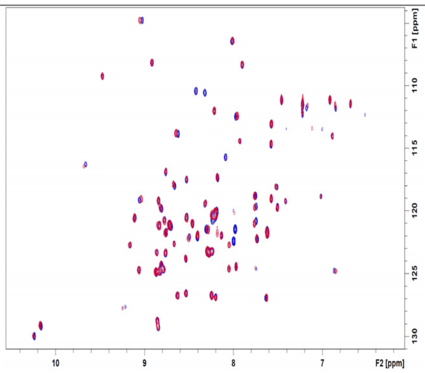
P1E4	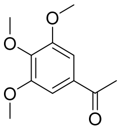	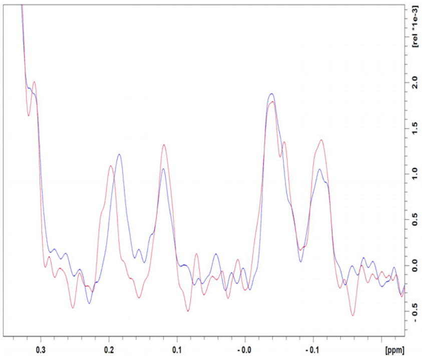	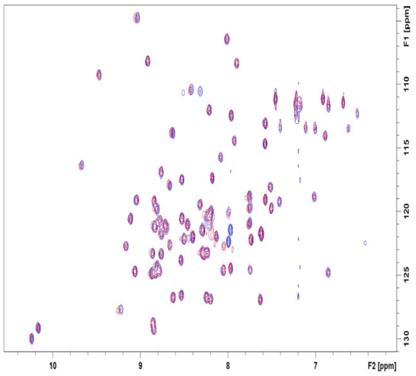

P1E5

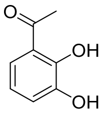

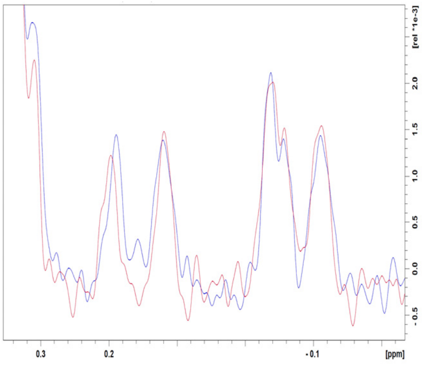

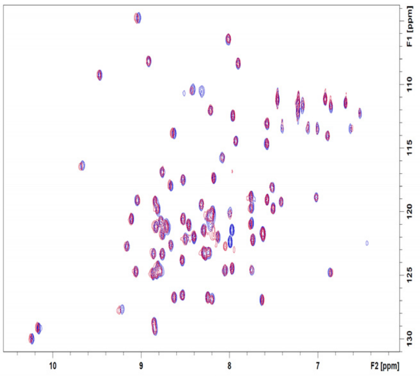

P1E9

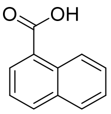

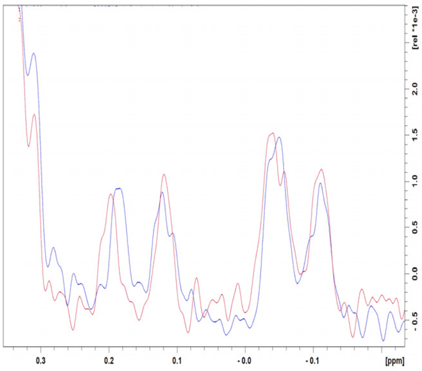

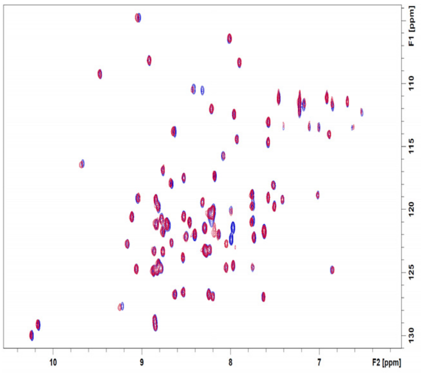

P2B7

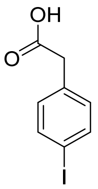

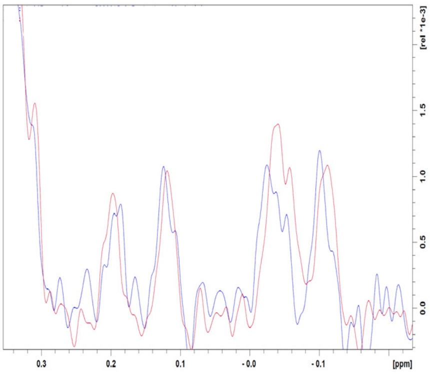

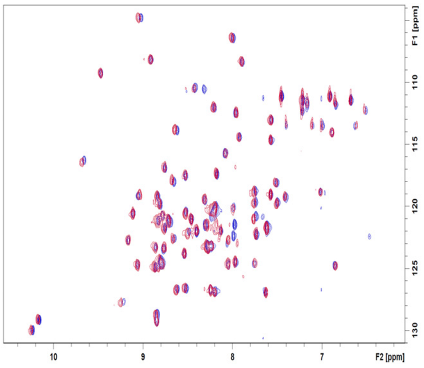

P2D9

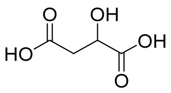

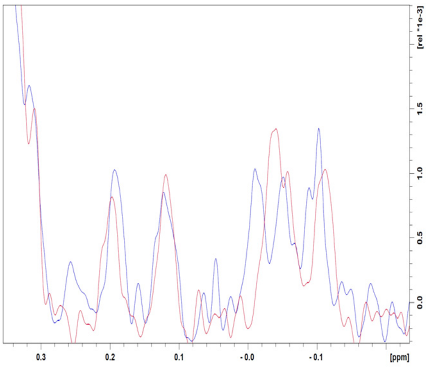

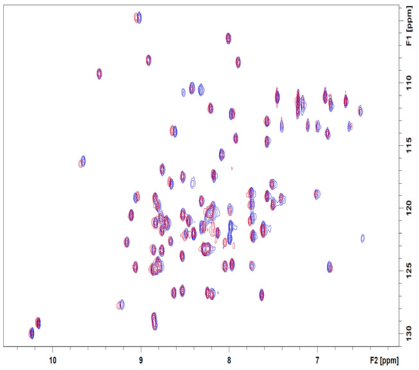

P2F7

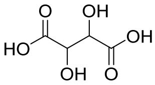

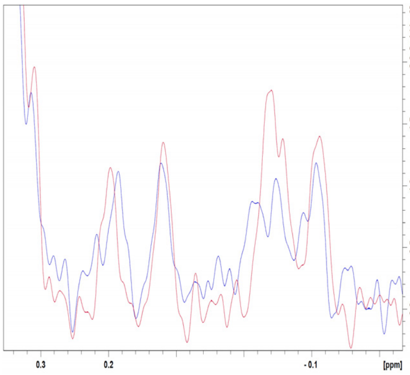

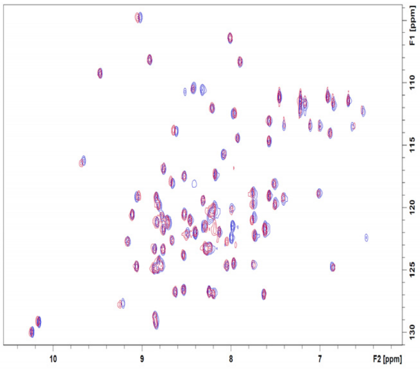

P2H1

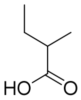

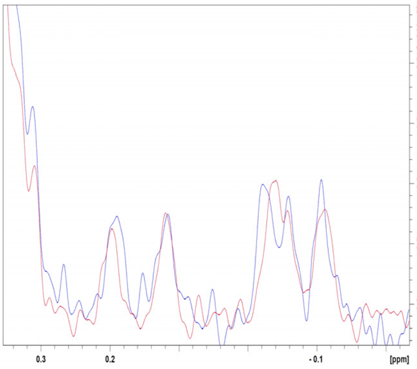

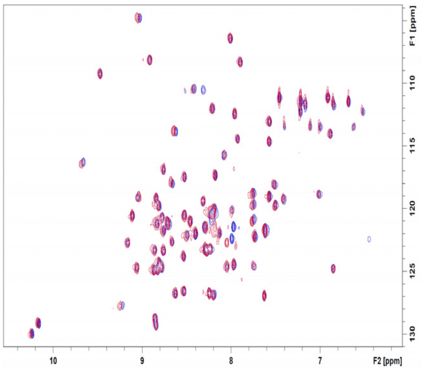

P2H4

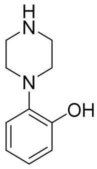

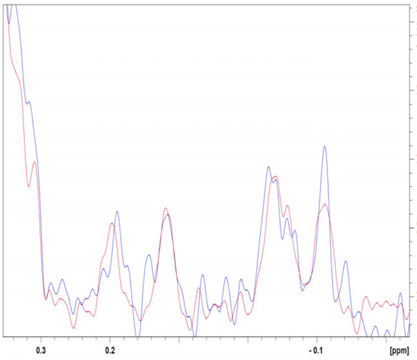

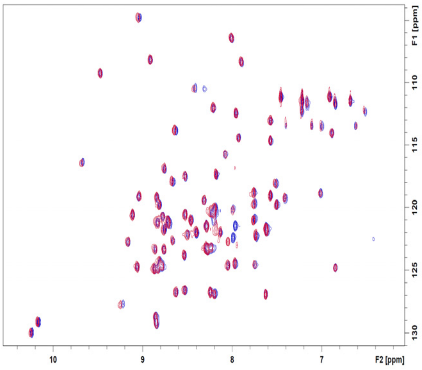

P4B10

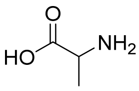

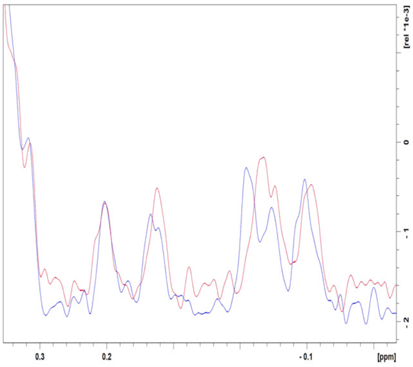

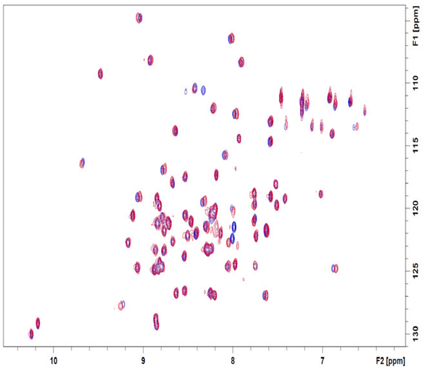

P4B11	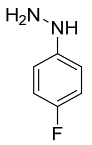	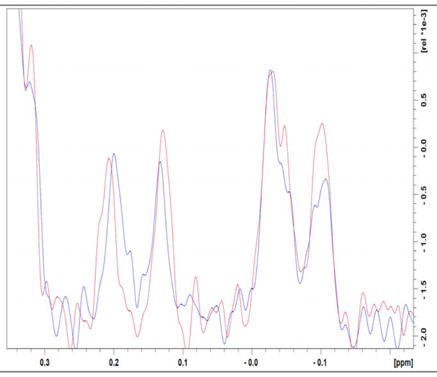	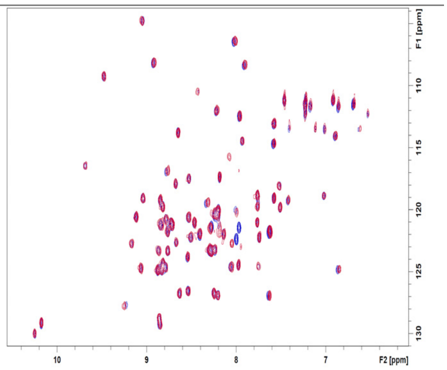
P4G7	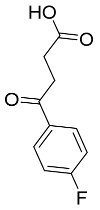	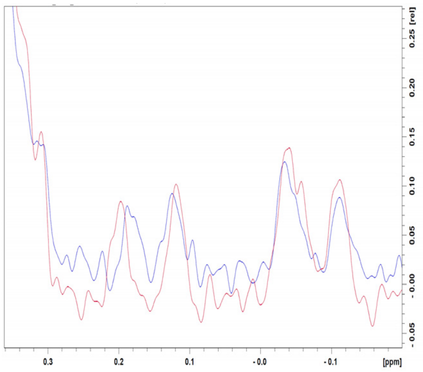	no data
P4G8	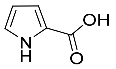	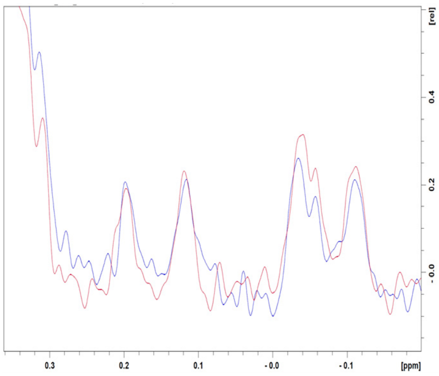	no data
P4G9	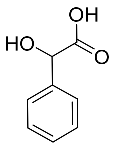	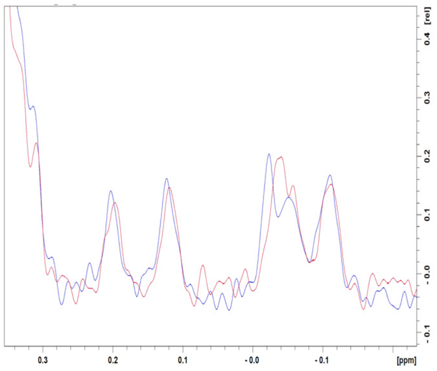	no data

P4G10

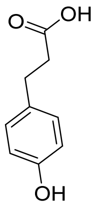

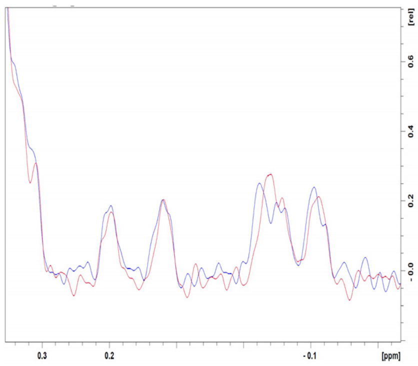

no data

P4G11

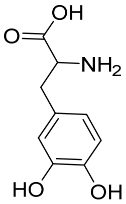

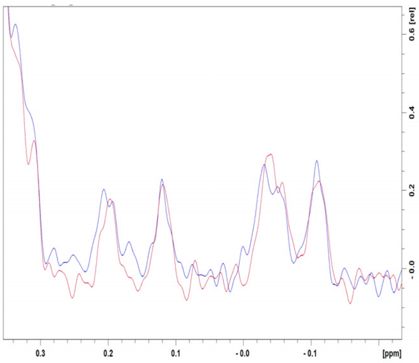

no data

P4G12

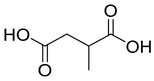

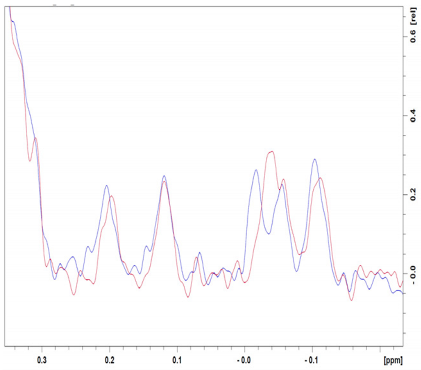

no data

P4H1

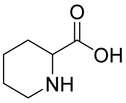

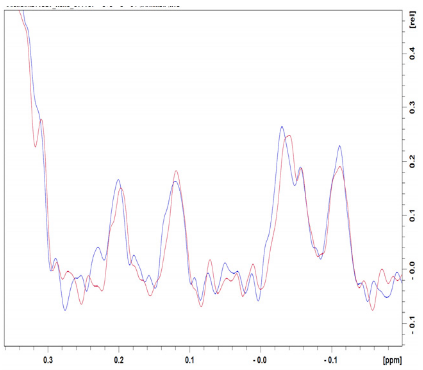

no data

P4H2	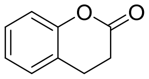	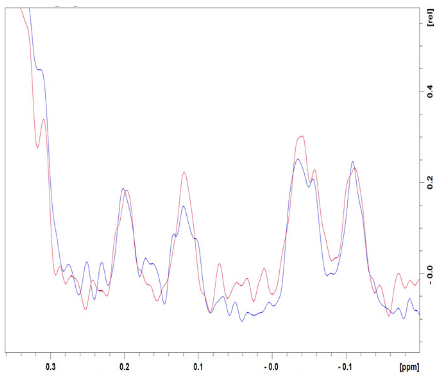	no data
P4H3	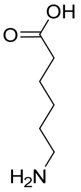	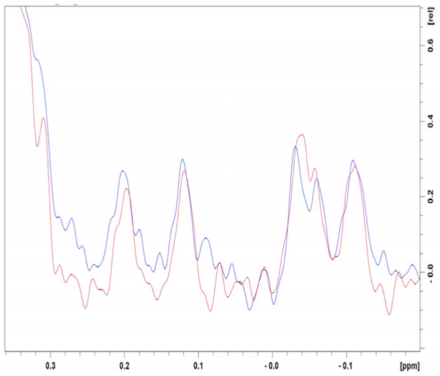	no data
P4H4	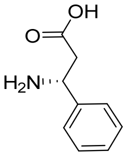	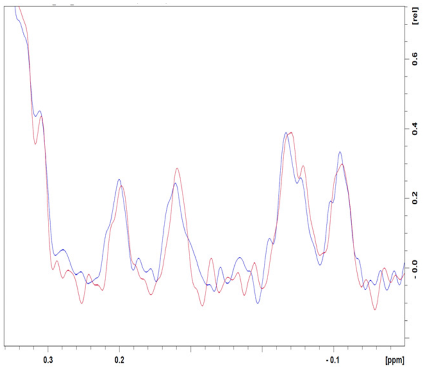	no data
P4H5	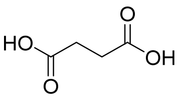	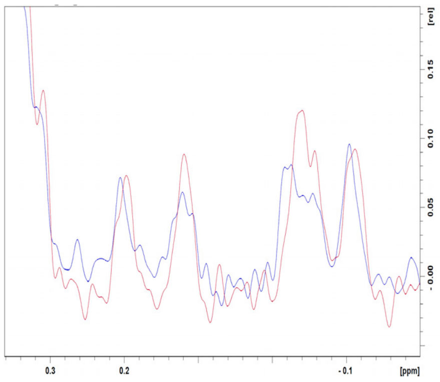	no data

P4H6	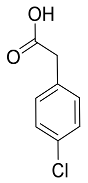	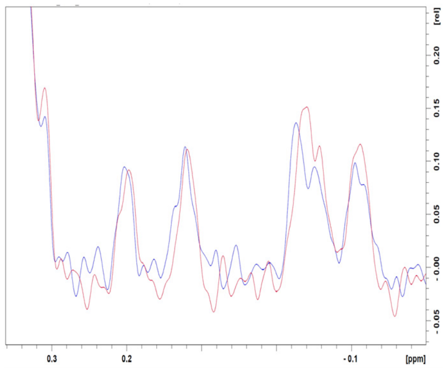	no data
P4H7	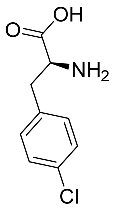	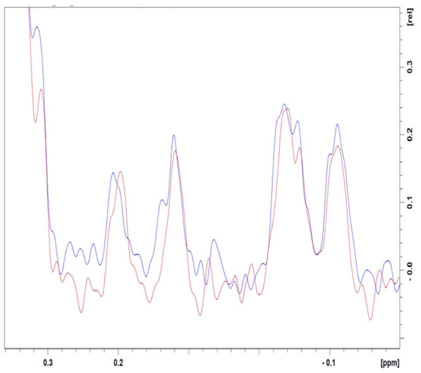	no data
P4H8	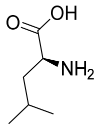	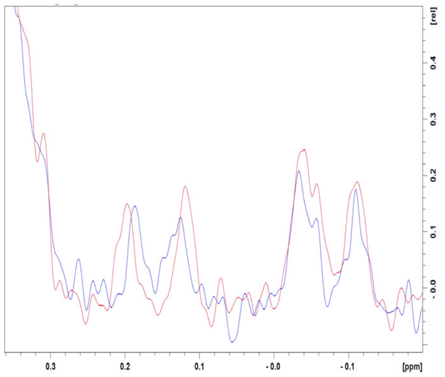	no data
P4H9	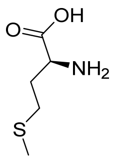	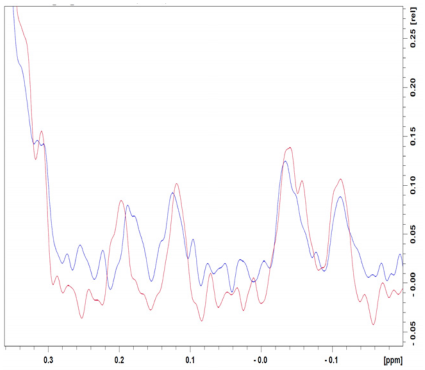	no data

P4H10	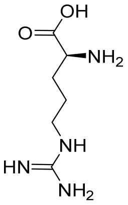	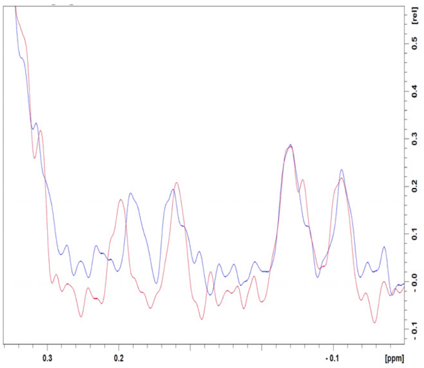	no data
P4H11	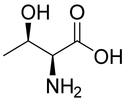	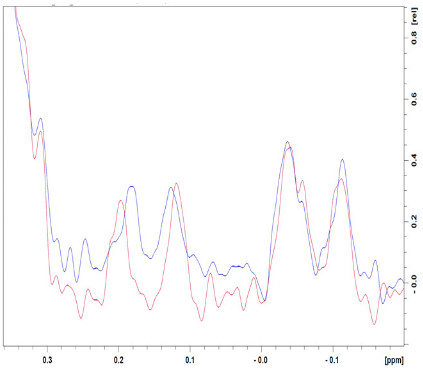	no data
P4H12	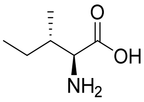	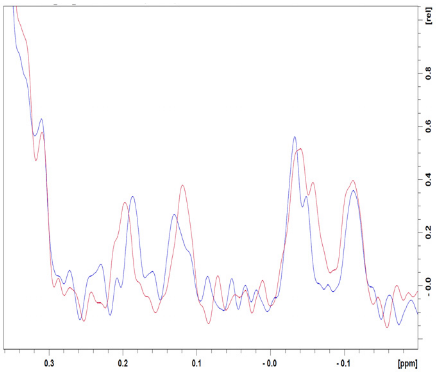	no data
